# Factors Affecting the Synthesis of Autonomous Sensors with RFID Interface

**DOI:** 10.3390/s19204392

**Published:** 2019-10-11

**Authors:** Mariusz Węglarski, Piotr Jankowski-Mihułowicz

**Affiliations:** Department of Electronic and Telecommunications Systems, Rzeszów University of Technology, Wincentego Pola 2, 35-959 Rzeszów, Poland

**Keywords:** RFID technology, semi-passive transponder, sensor, sensor network, autonomous sensor node, antenna, chip parameter, interrogation zone, ink-jet printing technology

## Abstract

A general view on the problem of designing atypical battery-free, autonomous semi-passive RFID transponders-sensors (autonomous sensors with RFID interfaces) is presented in this review. Although RFID devices can be created in any of the electronic technologies, the design stage must be repeated each time when the manufacturing processes are changed, and their specific conditions have to be taken into consideration when modeling new solutions. Aspects related to the factors affecting the synthesis of semi-passive RFID transponder components on the basis of which the idea of the autonomous RFID sensor was developed are reflected in the paper. Besides their general characteristics, the operation conditions of modern RFID systems and achievements in autonomous RFID sensor technology are revealed in subsequent sections—they include such issues as technological aspects of the synthesis process, designing antennas for RFID transponders, determining RFID chip and antenna parameters, creating the interrogation zone IZ, etc. It should be pointed that the universal construction of an autonomous RFID sensor, which could be use in any application of the automatic object identification system, cannot be developed according to the current state of the art. Moreover, a trial and error method is the most commonly used in the today’s process of designing new solutions, and the basic parameters are estimated on the basis of the tests and the research team experience. Therefore, it is necessary to look for new inventions and methods in order to improve implementations of RFID systems.

## 1. Introduction

Radio Frequency IDentification (RFID) systems have been used for years in many socio-economic activity areas and now they are more and more frequently being used instead of the popular bar codes [1]. The appreciable dissemination of this technology primarily results from the enormous progress in the electronics, as well as the advancement in better understanding of the phenomena that determine the possibility of exchanging information and transferring energy by electromagnetic waves. On the other hand, the continuous reduction in production costs of a unit RFID tag and the potential of integration RFID transponders with sensors of various physical magnitudes allows one to introduce this solution to contemporary developed areas of human activity, being the fastest growing markets ones like Fast Moving Consumer Goods (FMCG) [2] and the Internet of Things (IoT) [3]. Studies are also conducted in other aspects such as inventing transponder constructions that are easy to integrate with marked objects [4,5] (e.g., textile products [6]), elaborating localization and navigation systems in intelligent buildings, [7] creating autonomous, battery-free and wireless sensor networks [8] or detecting cracks of large-scale structures in the structural health monitoring (SHM) [9].

**Figure 1:**
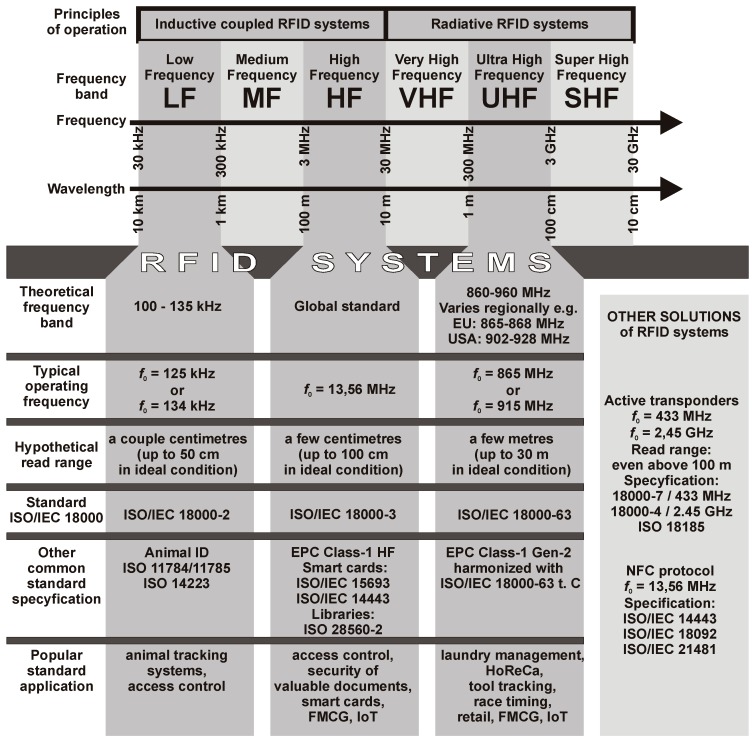
Basic information about RFID systems.

The internal structure of RFID devices (both read/write devices, RWD—usually called RFID readers or interrogators—as well as transponders, usually called as RFID tags) depends on many factors [1]. It is mainly determined by the frequency band (LF, HF or UHF) and operating frequency *f*_0_, due to various physical phenomena that constitutes the basis for energy transfer and information exchange in the used spectrum of electromagnetic waves (Figure 1). Part of the construction requirements are directly derived from specifications of communication protocols [10,11] but especially from conditions of desired RFID system implementation:

- the type of objects for which transponders are designed [12,13];
- the need to supplement transponders with additional functions (i.e. detection or measurement of different physical quantities, power management, possibility of obtaining energy from the environment) [14,15];
- desired read range which mainly depends on power supplying RWD output and shape of antenna system but also on sensitivity of input circuits (assuming constant conditions of radio wave propagation) [16,17,18];
- used electronic circuits (chips, microcontrollers or other microelectronic structures) which are often included in standards created by RFID industry (e.g., MIFARE, ICODE, HITAG—NXP Semiconductors, Eindhoven, Netherlands);
- assumed immunity to environmental hazards, but also costs, flexibility, designed way of integration with marked objects [19].

It should be noted that only the basic factors that influence the synthesis of RFID devices and that are discussed in the review are mentioned above. This shows how complicated the process of developing new devices of RFID systems is when high object identification efficiency is required.

A large part of the research works conducted by the authors was devoted to identifying the technological conditions and usefulness of available electronic components in the synthesis of semi-passive RFID transponders. The concept of the autonomous RFID sensor was developed on the basis of these considerations. Among others, four main parts can be indicated in constructions of this kind of device: antenna with matching circuit, chip (Integrated Circuit—IC), sensor/transducer and energy harvester/energy storage. Since the protocols for exchanging digital data between transponders and an RWD device are highly complicated, RFID chips are manufactured as ICs in semiconductor technology. On the other hand, it is very hard to integrate and reduce sizes regarding the rest of components (antennas and sensors) due to the principles of their operation. They can be fabricated using one of the typical electronic technologies (on rigid or flexible substrate, such as a Printed Circuit Board—PCB), thick-film or low temperature co-fired ceramic (LTCC) spatial structures, other hybrid structures combined with e.g., thin-film, screen or ink-jet printing elements, etc.) [20,21]. Then, all parts of the RFID sensor have to be connected together by one of the IC bonding methods (e.g., electrically conductive adhesive, wires or standard soldering) [22]. 

The paper is organized in such a way as to present the complete synthesis cycle of autonomous sensors with RFID interface in subsequent chapters. First (Section 2), the elaborated development board of the battery-free autonomous semi-passive RFID transponders-sensors is presented as a research object and the main problems encountered when synthesizing the device are highlighted. Next (Section 3), the possible constructions of electronic circuits tailored to assumed functionalities of different RFID system implementations are analyzed in detail. Since energy harvesting is one of the most important components in a battery-free device, the problem of designing the Radio Frequency (RF) frontend is presented in a separate section (Section 4). Prior to elaborating inductive circuits, it is necessary to know the factors affecting the results of technological processes as well as the properties of materials used both in tag construction and in the structures of marked objects. The research aspects found in these synthesis stages are presented in Section 5 and Section 6, respectively. Then, based on a general model of the RF frontend for the RFID transponders (Section 7) and the knowledge of how to determine the input parameters of RFID chips (Section 8), it is possible to manufacture effective RFID antennas. The methods of confirming RFID antenna parameters are suggested in Section 9. The final stage of the transponder synthesis process dedicated to a given application is to determine the read range or the interrogation zone and this aspect is described in Section 10. The last two Section 11 and Section 12 are devoted to the authors’ inventions protected by patents: RFIDtex textronic products and intelligent systems of localization and navigation by using RFID technology.

## 2. Development Board of Autonomous RFID Sensor

**Figure 2:**
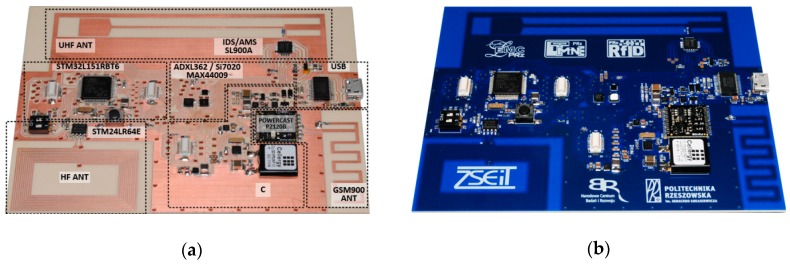
Demo boards of autonomous RFID sensor: (**a**) Test version manufactured in authors’ scientific HYBRID Laboratory; (**b**) Production version manufactured in ELMAK Ltd Company (Rzeszów, Poland).

The design issues of the battery-free autonomous semi-passive RFID transponders-sensors are presented on the basis of research that was conducted during the development of the demonstration board of the autonomous RFID sensor [23]. The development board (Figure 2a) is designed for operation in two frequency bands: HF (according to the ISO IEC 15693 protocol) and UHF (EPC Class 1 Gen 2, ISO/IEC 18000-63), and for harvesting additional energy from one of common telecommunications system (GSM 900). The project is based on HF STM M24LR64E (STMicroelectronics, Geneva, Switzerland) and UHF AMS SL900A (AMS AG, Premstaetten, Austria) chips equipped with serial data links (I2C and SPI, respectively) that allow communication with the microprocessor measuring module with implemented 3-axis MEMS accelerometer (ADXL362, Analog Devices, Norwood, MA, USA), humidity and temperature sensor (Si7020, Silicon Labs, Austin, TX, USA) and lighting sensor (MAX44009, Maxim Integrated, San Jose, CA, USA). Autonomous operation of the device is obtained using the Powercast P2110B RF frontend (Powercast, Pittsburgh, PA, USA) [24], which acquires energy from the electromagnetic field in the 930–975 MHz band (GSM 900). The device also includes a supercapacitor with an appropriate charging, power management and control system based on a 32-bit microcontroller with ARM core (STM32L151RBT6, Low Power type). The demonstrator was assembled on the technology line of the Elmak Ltd (Rzeszów, Poland) electronic company (Figure 2b) and was prepared as the development kit for potential designers of RFID systems. 

It should be mentioned that studies of the consumer market were one of the stages in developing the kit of a battery-less RFID sensor. These led to the identification of the need to implement such systems and factors influencing decision making in this area [25]. Based on the obtained results, the management style, trust in subordinates, leadership in terms of change, organization of work, commitment to work, level of motivation to undertake new tasks, orientation on organization development and cooperation with others are the main factors that prompt Polish managers to implement unconventional RFID devices [26]. On the other hand, features of the new product (innovative autonomous semi-passive RFID transponder), its attractiveness and quality along with implementation costs, price and return on investment are emphasized in Polish enterprises. Managers with great experience in implementing investments and using RFID systems particularly appreciate the advantages of the new product and represent a more optimistic investment approach than the managers inexperienced, whose are afraid of the cost and risk pertinent to investment decisions [27].

The first step prior to synthesizing the RFID sensor is to select suitable electronic circuit components (chips, transducers, energy harvesters) in order to achieve the assumed functionalities and to designate input data to numerical models of RF frontends (consisting of an antenna, matching circuit, chip input circuits, etc) (Section 3). Since the RFID market of chips with acquisition modules is not very mature yet, this task is extremely difficult and alternative equivalents consisting of a few discrete elements have to be elaborated. It is very hard to build an autonomous RFID sensor based just on commercial ICs because they only partially fulfill the established assumptions. One of a few such products is the AMS SL900A chip designed to operate in semi-passive transponders (although it can also work in passive mode) which is equipped with additional functions and extended user memory. A problem with designing a supplying unit that works effectively at a very low level of voltage generated at antenna terminals is also the important challenge [28] (Section 4). The best case would be if this block was supported by additional energy storage (e.g., supercapacitor) as well as an appropriate management algorithm of device activity to ensure its stable operation in a weak electromagnetic field.

During the design process of RFID sensor antennas, attention should be paid to the properties of the materials used both in the tag construction as well as in the structure of marked objects [29] (Section 5). Since electronic transponders should always be cheap and easy to integrate with marked objects, the ink-jet printing of conductive materials on elastic substrates seems to be one of the best prospects for high volume production [30,31]. On the other hand, in the laboratory, the synthesis of this type of electronics is burdened by the great number of problems which must be solved. It turns out that transponders dedicated to harsh environmental conditions, which are the most often implemented on the basis of commonly known printed circuit boards, can be made much easier than typical flexible tags. The availability of PCB technology and the simplicity of creating test models at the design stage make it ideal for conducting experimental works. Thus, the PCB has been used to prepare test devices that are presented in the review but research efforts regarding technological aspects have been put especially to constructions made by ink-jet printing. Since the significant problem is with access to information about values of basic parameters, e.g., of substrates (mainly, to information about relative permittivity *ε_r_* and loss tangent tg*δ*, because in manufacturers’ datasheets they are often omitted or are given in limited frequency bands), it is necessary to use one own’s test procedures in order to determine these quantities [23] (Section 6).

One of the major factors for effective implementation of RFID system is the quality of the antenna system: antenna of RWD, RF harvester and transponder [32,33]. The design process of these induction circuits is particularly complicated, because the classical theory cannot be applied (Section 7). The interactions with other antennas or discrete elements integrated on one substrate as well as with environment have extreme impacts on the elaborated construction. Further, the antenna quality factor should be relatively high, and the impedance should exactly correspond to the parameters of the chip input RF frontend (including a matching system) in order to ensure the possibility of harvesting energy from the weak electromagnetic field (Section 8). RFID sensors (e.g., the AMS SL900A) with *V_OUT_* output loaded by external circuits have to cooperate with a specially designed antenna because the standard constructions block the operation of the energy harvester in the chip [32,34,35]. The problem can be solved using special solutions of the matching circuit (for example constructions based on elements that block the constant component of current) which affect the target shape of the inductive loop (Section 9). The abovementioned requirements are in contradiction with the need to minimize the dimensions of the designed autonomous RFID sensors. 

On the basis of worked out know how, designers can determine the read range (maximal distance between centers of the tags and RWD antenna) and the interrogation zone as the main parameters that describe efficiency of every RFID system (Section 10). The above described research activities in the field of the RFID technology are reflected in the development of a new construction including inventions protected by patent, such as RFIDtex textronic products (Section 11) or intelligent localization and navigation systems (Section 12). 

## 3. Autonomous RFID Sensor

**Figure 3:**
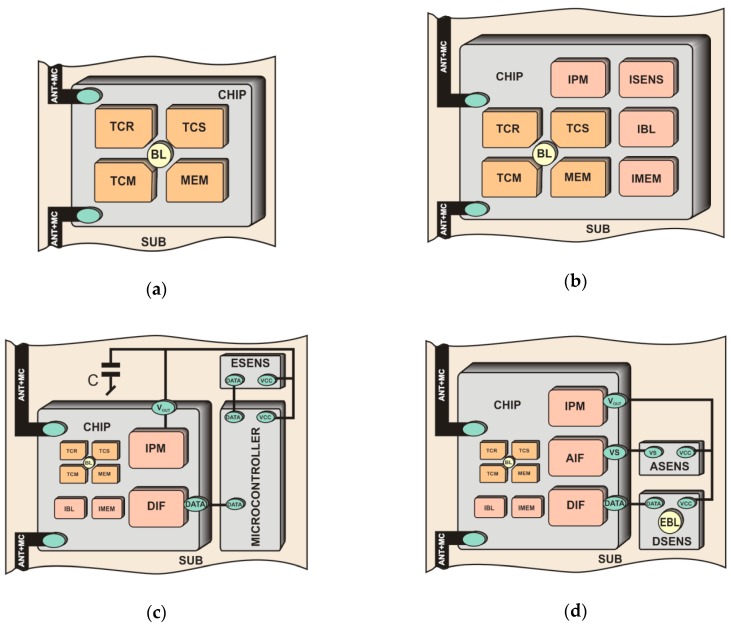
Different concepts of RFID sensors: (**a**) A typical passive transponder; (**b**) Passive transponder with sensor on chip; (**c**) Digital platform with passive transponder; (**d**) Passive transponder with analog or digital sensor interface. TCM, TCR, TCS—respectively: modulator, rectifier, voltage regulator at the chip’s input circuits; BL—digital part of the chip; MEM—basic data memory with UID (Unique Identification Number); IBL—internal digital data acquisition block; ISENS—internal transducer of temperature, pressure, humidity, etc.; IMEM—extended data memory on chip; IPM—power supply management circuit/internal RF harvester; AIF—analog sensor interface/input of analog-to-digital converter; DIF—digital sensor interface (e.g., SPI, I2C); ASENS—sensor with analog output; DSENS—intelligent sensor; ESENS—external sensor, typically intelligent but also with analog output; SUB—RFID tag substrate; ANT—antenna; MC—matching circuit; VS—analog signal from sensors; DATA—digital signal from sensors.

Passive RFID transponders are the most widespread and accessible construction in the practice of automatic identification (Figure 3a). They are composed of two main parts: the chip and the antenna attached to the chip terminals. The energy needed to power the electric circuit of the transponder is obtained from the electromagnetic field generated by the RWD antenna and it is usually enough only to maintain the process of data transmission with the interrogator. Nevertheless, with the progress in micro- and nanotechnology of electronic structures, there is the possibility to design more and more energy-saving integrated circuits. The ultra-low power RFID chips can be equipped with additional atypical functions, especially that mechanisms allowing for expanding protocols (acquired data can be pass to RFID reader by load them to the extended memory of the chip) are provided by existing communication standards (e.g., in the UHF band: Electronic Product Code Class 1 Generation 2 [36], harmonized with ISO/IEC 18000-63 type C [37]). 

In research laboratories all over the world, CMOS circuits integrated with transducers of different physical quantities are under intense investigation (RFID chips with internal sensors of temperature [38,39], pressure [40], humidity [41], etc.) where the most important problem is to cope with the energy deficit and measurement precision. Thanks to the close technological conjunction between RF and measuring blocks integrated on a common chip, designed transponders with sensing functions can be optimized and significantly miniaturized, what is especially important in low-cost applications (Figure 3b). Nevertheless, such RFID sensors provide substantially lower accuracy and range of measuring values than typical MEMS encapsulated in stand-alone integrated circuits. This is due to the fact that the supply power is provided by a chip rectifier which harvests energy from unstable and unpredictable electromagnetic field (supply noise significantly influences the sensor accuracy [42]). On the other hand, transponders can include stand-alone sensors (with analog or digital output) that are attached to low power microcontrollers with RFID interfaces. They can be based on two different constructions: a RFID frontend integrated with a low power microcontroller [39,43] (Figure 3c) or on a RFID chip which provides a voltage output *V_OUT_* for external circuitry [44,45] (Figure 3d). Both of them suffer from a lack of enough energy and can work only in a strong electromagnetic field. Although the construction of passive RFID sensor is feasible and useful in many applications, it cannot operate autonomously without the activity of a read/write device.

Besides integrating sensors into the electronic circuits of tags, there is also possibility to influence the physical principles of operations inherent to RFID systems [46]. It is feasible to use materials that are sensitive to a given physical quantity and to use them in the construction of the transponder in order to change the chip activity, operating frequency [47] or attenuation of backscattered signal [48]. Such devices are called usually tag antenna-based sensors [49]. A great example in this scope is structural health monitoring that is used to detect corrosion in civil and industrial sectors such as transportation, marine construction, public infrastructure, etc. The passive antenna-based RFID sensors provide the possibility of cheap, wireless and log-term monitoring that can be permanently embedded in different type of structures. The important problem is to deal with read range or interrogation zone IZ (both parameters enable the practical estimation of RFID system effectiveness and they are explained in details in Section 10) as well as metal obstacles, conductive materials or fluids that influence inductive and capacitive parameters of the devices. For example, conductivity and permeability changes of a corrosion area can be detected by LF RFID sensors [50]. The devices can be used for non-destructive tests even at high temperature values [51].

**Figure 4:**
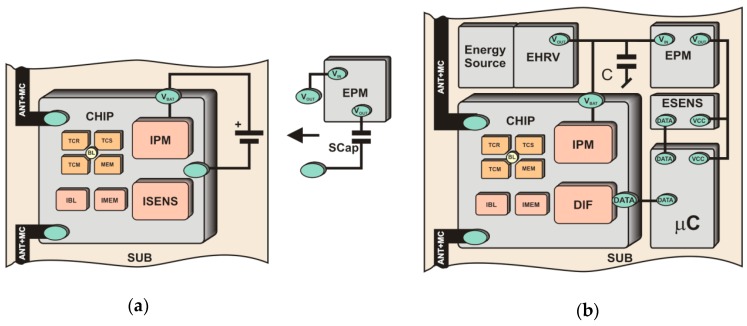
Different concepts of autonomous RFID sensors. (**a**) Construction based on semi-passive transponder; (**b**) Construction based on semi-passive transponder and external energy harvester. EPM—external management circuit of power supply and battery charging; EHRV—external energy harvester; SCap—supercapacitor; µC—microcontroller; Energy Source—transducer of light radiation, thermal radiation, mechanical vibrations, electromagnetic field of public radiocommunication systems, etc.

The additional functions are more often implemented in semi-passive transponders where a replaceable or non-replaceable power source (usually a battery) is built-in (Figure 4a). It should be noted that in this case the battery source is not used to activate the radio communication frontend of the chip. Since the transponder antenna does not emit an electromagnetic field but only modifies it by changing the impedance, the RF frontend is powered by energy harvested from radio waves. The extra energy is used to cover loads generated by digital circuit and any other functional blocks that are added to tags. However, it can be used to increase the chip sensitivity, which extends linear read range or enlarges spatial interrogation zone IZ. In addition, the constant and predictable power supply favors the trend of equipping semi-passive transponders with transducers of different physical quantities. Such an autonomous RFID sensor can operate (e.g., perform data acquisition functions) both in the interrogation zone as well as autonomously outside it (e.g., in the case when any read/write device is not active). Nevertheless, high energy demand generated by additional functional units and thereby the need to use an electrochemical power sources is so troublesome that they have not yet gained significant importance on the commercial market. The options of harvesting energy from the environment (Figure 4b) are described, in many literature considerations where light radiation [52], thermal radiation [53] or mechanical vibration [54] sources are mainly mentioned. The output power in these types of supply units [55] is large enough for permanently providing the modern digital systems with energy. An example of such a multimodal RFID sensor is described in [56]. It is generally based on wireless identification and sensing platform (WISP) [57] supplemented with two temperature sensors, a humidity sensor, a 3-axis accelerometer and an external 1 Mb EEPROM. The relatively high demand for power is just covered by the photovoltaic cell. The disadvantage of the construction, however, consists in the problem with integrating the additional energy harvester and the RFID tag in one common structure, especially when minimal size, low manufacturing costs and simplicity of connection with the marked object are main requirements.

The idea of the autonomous RFID sensor fits the need of searching for new solutions and their implementation in real automatic systems of object identification. It is also developed in the scope of using electromagnetic field generated by public radio systems (not by an RFID read/write device) to supply the RFID sensor [58]. This kind of energy source can provide only a small amount of power (e.g., working in the GSM band 0.9 GHz or 1.8 GHz—up to 0.1 μW/cm2, WiFi 2.4 GHz—up to 0.01 μW/cm2 [55,59]) and the use of highly energy-efficient electronic circuits and effective antenna frontends are required. Thus, it is the reason why such an approach has not yet been applied in the common practice of designing RFID transponders. However, it gives the possibility to integrate all elements in only one technological process. 

The implementation of the autonomous RFID sensor concept is possible thanks to the use of chips that incorporate a wireless and wired communication interface as well as the possibility of gathering energy from the RFID field and providing harvested power to external electronic circuits. These ICs belong to the new solutions that are currently being developed in scientific and industrial research laboratories and their final designs are slowly becoming widely available on the market. For example, in RFID systems of the UHF band (860–960 MHz), operating in accordance with the requirements of the second generation electronic product code [35] which is standardized by the ISO/IEC 18000-63 [36], there are accessible such products as the AMS SL900A [60], Farsens ROCKY100 (Farsens, San Sebastián, Spain) [45], EM4325 (EM Microelectronic, Colorado Springs, CO, USA) [61] and Cypress WM72016-6 (Cypress Semiconductor, San Jose, CA, USA) [62]. They are dedicated to battery-assisted semi-passive transponders and their selected parameters are listed in Table 1. Moreover, they enable harvesting energy from the electromagnetic field of the RFID system and thus providing the possibility of developing the RFID sensor that could work at the presence of an active read/write device. By equipping the transponder with supercapacitor (Figure 4a) it is possible to perform measurement tasks outside the interrogation zone but only by a short time (depending on the capacitance of the supercapacitor) and only in pulse regime (in order to save energy). It gives the idea of semi-autonomous RFID sensor. Nevertheless, all chips can be also powered from external sources (battery-assisted mode) which allows them to work autonomously (it fulfills the idea of the autonomous RFID sensor) in a typical semi-passive mode.

The significant structural differences (Table 1) are primarily visible in terms of user memory size and the ability to support peripheral devices (transducers/sensors). Some of the chips can measure the temperature of the internal semiconductor structure and thus can act in passive mode as passive transponders/sensors (Figure 3b). Since they are usually equipped with a serial data interface (SPI or I2C), they can also cooperate with intelligent digital (microprocessor) systems with additional transducers of various physical quantities [63]. But then, the semi-passive construction has to be developed since the amount of energy harvested from the electromagnetic field of the RWD is not sufficient (Figure 3c,d).

Considering the need to register as much information as possible about the environment of an electronically marked object, the WM72016-6 chip seems to be the most useful one. It provides almost 16 kb of internal memory intended for user data. On the other hand, there is no built-in temperature sensor in its structure that could be useful in the conceptual work and in addition—it is not possible to connect analog transducers directly to the external terminals. The ROCKY100 circuit which is an improved version of its predecessor ANDY100 has a similar structure and application utility. However, it should be emphasized that only 1008 b of user memory are available in this product. The AMS SL900A and EM4325 chips include internal temperature sensors that can be operated from both the wired (RFID) and wireless (SPI) interface. The first one can also support an external temperature sensor with a wider range of operation (−40–125 °C). Furthermore, this system has two 10-bit analog-to-digital converter inputs that can be used for connecting transducers of various physical quantities with analog outputs. And important advantage of the AMS SL900A is the user memory of 8416 b that is available on the chip, which is more than double the size of the EM4325.

The comprehensive analysis of conditions for developing the semi-autonomous RFID sensor with function of harvesting energy from the electromagnetic field generated by the RWD is given in [58]. Primarily, possible concepts of the developed device are proposed as well as pros and cons of each structure are indicated with comparison with platforms described in the literature (WISP [57], SPARTACUS [64] or XT01 Tag [65]). The constructions are based on both microprocessor circuits cooperating with an RFID chip supplied with power derived from a read/write device and radiocommunication systems as well as are built using only an RFID chip supported by additional energy storage (e.g., in supercapacitors). 

**Figure 5:**
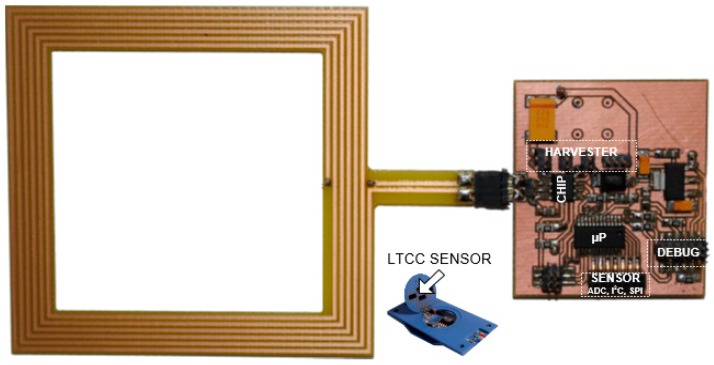
Practical implementation of semi-autonomous RFID sensor operating in the HF band, with LTCC pressure transducer.

The construction dedicated to the HF band (*f*_0_ = 13.56 MHz) [58] (Figure 5) is based on the dual-port STM M24LR64E-R which complies with the standards: ISO/IEC 15693, ISO/IEC 18000-3 mode1 [66] (a similar solution can be implemented on Ramtron F-RAM memory (Ramtron, Cypress Semiconductor, San Jose, CA, USA) for the UHF band in the frequency range of 860-960 MHz [67]). Since the allowable load of the *V_OUT_* output (output of internal energy harvester of the chip) is dependent on the intensity of the weak electromagnetic field at a point of the IZ, the special attention has to be paid to the energy balance of the acquisition system. The design of the quasi-autonomous RFID sensor is possible using external energy storage element (supercapacitor). It means that the RFID sensor can measure outside the IZ but only by short time in a pulse mode.

**Figure 6:**
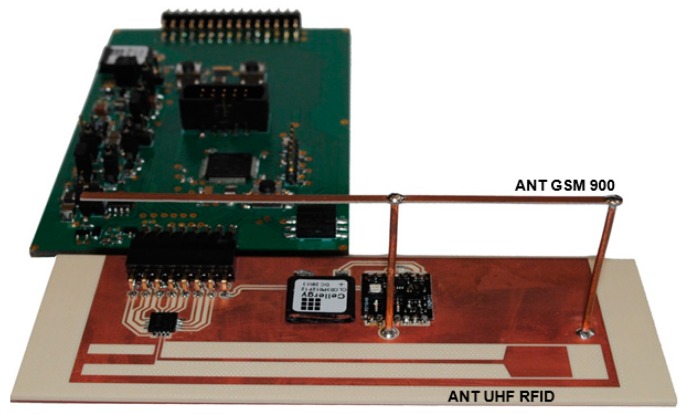
Practical implementation of autonomous RFID sensor operating in the UHF band. ANT GSM 900—antenna of harvester gathering energy from electromagnetic field generated by telecommunication system GSM 900, it is connected to Powercast P2110 module; ANT UHF RFID—antenna of RFID system operating in the UHF band, it is connected to AMS SL900A chip.

The fully autonomy of the electronic tag can be obtained by supplementing the structure of semi-passive RFID transponder with a block that harvests energy from the environment [59]. The model of developed construction is designed using the AMS SL900A chip (Figure 6) [52] operating in the UHF band (a similar solution can be implemented in the HF band using the SL13A chip [68] or its the newest version AS39513 [69]; it can also be made in limited range on the previously mentioned STM M24LRxxE-R or Ramtron FRAM chips). The advantage of the AMS SL900A is that it has a built-in analog-digital converter, so there is no need to add an external microprocessor system that could manage the data acquisition process (however, the microprocessor acquisition system is implemented in the presented RFID sensor in order to conduct tests on load impact on the devices performance). The energy necessary for providing to measuring blocks is obtained from the 925–960 MHz band (the operating band of mobile phone base stations—GSM900) using the Powercast P.2110 radio frontend [24].

## 4. Energy Harvesting in Autonomous RFID Sensors

**Figure 7:**
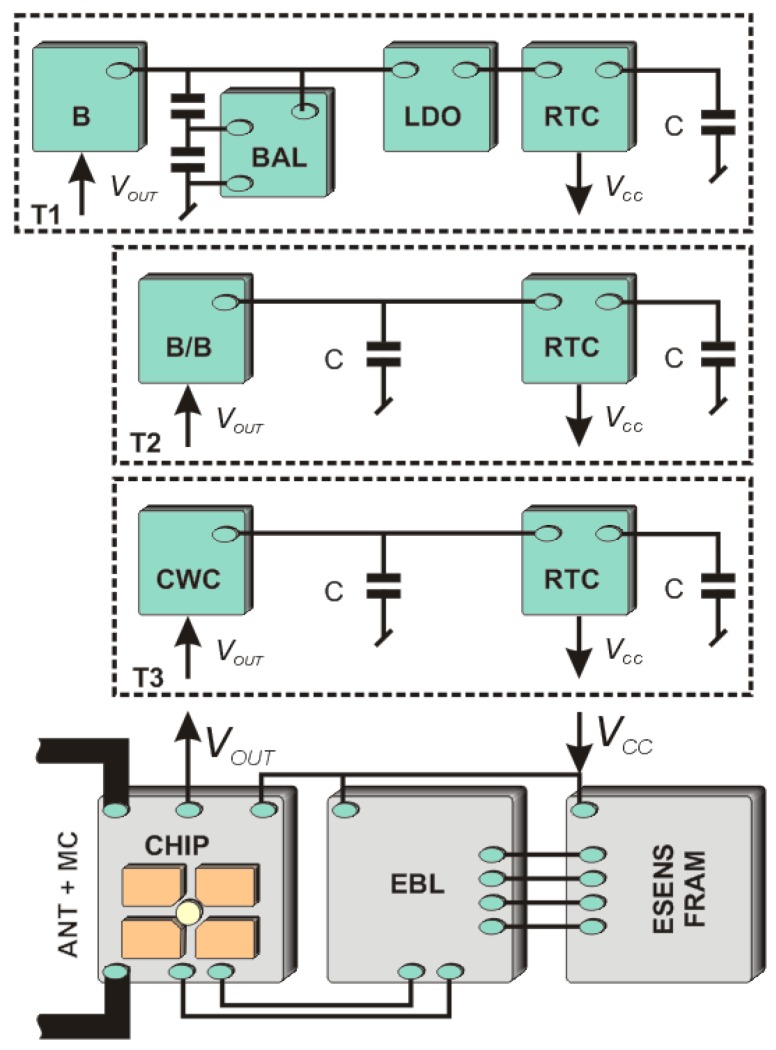
Considered model of data acquisition systems for quasi-autonomous RFID sensor. T1, T2, T3—versions of the energy conditioning and storage system; CWC—voltage regulator; B/B—Boost/Buck voltage converter LTC3531; B—Boost voltage converter TPS61220; LDO—Low-Drop voltage regulator TPS78318; BAL—supercapacitor charging circuit; RTC—real time clock with energy supervisor AM1815; FRAM—ferromagnetic random-access memory MB85RS1MT; EBL—external digital data acquisition block; C—capacitor or supercapacitor.

Aspects related to designing energy-saving microprocessor systems with data acquisition functions in battery-free quasi-autonomous RFID sensors of the HF band can be found in [28]. The problems concerning the design of the supply circuit (T1, T2 and T3, Figure 7) that gathers, processes and stores energy derived by the internal harvester of the RFID chip as well as the possibility of providing conditioned energy on the external output *V_OUT_* is discussed by the authors in this paper. 

There is an increasing number of chips with dedicated output (*V_OUT_*) for suppling external circuit with the voltage generated by the internal energy harvester. Generally, the voltage *V_OUT_* ranges from about 1.5 V to 4.5 V (e.g., for M24LR16E) and it is depended on the intensity of the electromagnetic field *H* where the tag is located. The chip’s harvester starts working when the field strength exceeds the minimum value of *H_min_*. This enables to activate sensing functions in transponder, but the sudden voltage decay has to be predicted in the management procedures. When considering semi-autonomous mode of the sensing node, beside the energy storage the time markers for measuring samples have to be also predicted in designing concept of supplying block. Based on conducted analysis of energy balances for several different constructions, a system with an external real time clock (RTC) having the power management function seems to be the best choice [28]. However, RTC blocks can be found on typical microcontrollers but when it is used the energy consumption is higher. 

A ceramic capacitor or a supercapacitor can be implemented as the energy storage device. The first is characterized by low capacity but high charging rate and thus a short time of reaching the nominal voltage after full discharge. It means that the system starts quickly as only transponder appears in the IZ but also its activity can last only short time after disappearance of the electromagnetic field.

With appropriate selection of a high capacity supercapacitor, the operation time of the RFID sensor in the quasi-autonomous mode can be extended significantly. Its use relates to the necessity of delivering much more energy before the minimum level of operating voltage is reached. In the case of dynamic changes in the electromagnetic field generated by the RWD antenna it is not possible to provide stable power conditions by applying this construction. In addition, a leakage current and acceptable operation voltage (about 2 V) of available supercapacitors which is lower than the maximum output voltage on *V_OUT_* (about 1.5–4.5 V) are also a significant problem. In the simplest case, it can be solved by using a voltage stabilizer (T3 model; Figure 7), nevertheless, the use of a DC/DC converter of "Buck/Boost" type (T2 model) slightly improves the charging efficiency. The highest ratio of the useful energy to the total energy provided to the storage element is obtained using the serial capacity connection (T1 model). Such a solution requires an extra voltage balancing circuit due to parameter dispersion of produced supercapacitors. In the energy balance of proposed constructions, loads generated by additional conditioning blocks (CWC, B, B/B, LDO, BAL) should be considered each time.

It is also worth paying attention to ferromagnetic access random memories (FRAM), which are more and more often used in the practice of designing microprocessor systems. The FRAM is a kind of non-volatile memory with writing cycle time of about 2 µs that is much shorter value than in the case of standard EEPROMs or FLASHs (about 5 ms). Its use significantly affects the duration of software procedure that is necessary when measured samples are stored in a local memory of the RFID sensor. Thus, the duty cycle of the semi-autonomous work can be even 100 times shorter and most of it can be devoted to performing the measurement functions. Since the duty cycle can be shortened the energy consumption is also lower. 

**Figure 8:**
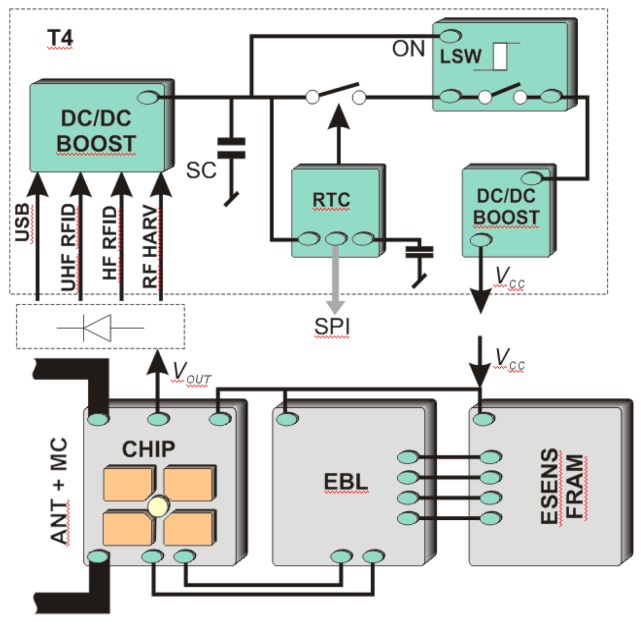
Diagram of advanced power supply circuit dedicated to fully autonomous transponder. DC/DC BOST—ultra efficient step up switching regulators Silicon Labs TS3310; SC—supercapacitor Cellergy CLC03P025F12; LSW—ultra low ON-resistance load switch with controlled turn on with hysteresis Texas Instruments TPS22934; RTC—low power real time clock Abracon AB1815.

The advanced power supply circuit dedicated to fully autonomous transponder is proposed in [23] and described as T4 in Figure 8. It allows the supercapacitor charging from three sources associated with each of the antenna circuits (Figure 2), even at very low intensity of the electromagnetic field (minimum operating voltage is 0.85 V with 0.02 V hysteresis). It also manages the measuring block, keeping it in an idle mode (only an additional RTC remains active all the time) every time when the accumulated amount of energy is insufficient to achieve assumed software algorithms. The two DC/DC step up converters that provide conditioned voltage, completely isolate loads from the energy storage capacitor (SC). They improve the efficiency of energy harvesting thus the supercapacitor can be fully charged even in the presence of weak electromagnetic field. The ON-resistance load switch supports managing process by keeping acquisition system (EBL, ESENS) from turning on till the time when the reservoir of energy SC reaches enough level. The real time clock is used to weak up sensors when short-time duty mode is set up. 

The contemporary state of the integrated circuit market has been considered when the energy balance of the demonstrator was analyzed. A great problem is self-discharging of the supercapacitors even when the all transponder’s blocks are turn-off or in sleep mode. It is due to leakage and quiescent current which occurs in all passive and active components. Thus, the permanent process of energy harvesting, e.g., from common radio transmitters is only the possible choice. However, the continuous progress in electronic technology ensures that practical implementation of such constructions will be easier.

## 5. Technology Factors Affecting Synthesis Process

The antenna is the main component of a RFID transponder which effectiveness is the most sensitive to the technology used in the manufacturing process. It can be realized in the PCB, LTCC, on ceramic or elastic substrates, using screen printing or other printing techniques adapted to the manufacturing electronic circuits. The chosen technology determines methods of mounting discrete elements (e.g., chip, matching passive component or sensor) to antenna terminals, e.g., by using electrically conductive adhesives, wire bonding or standard soldering. The compatibility of the techniques and methods used to fabricate electronic structures determines the performance of the obtained RFID tag.

The availability of printed circuit boards as well as the low cost and simplicity of creating test models on copper clad laminate at the design stage make this technology ideal for conducting experimental works. Nevertheless, research efforts regarding technological aspects have been put especially to constructions made by ink-jet printing, because this technique is predicted to be widely used in near future although it currently causes significant problems in RFID tag fabrication, especially in the research laboratory conditions.

Ink-jet printing is one of the least mature method, even though there are many advantages that predispose it to be used in high-volume production [70]. Using this technique, it is possible to print functional materials (conductive, dielectric and even semiconductor) and thus to manufacture RFID transponder components directly on the labeled product, their packaging or substrate (plastic, paper, ceramic, metal, etc.) [71]. This is an additive method thus the amount of production waste is much lower than in other technologies, which brings both environmental and economic benefits. Its huge advantage results also from the fact that it belongs to the group of printing techniques perfectly suited for roll-to-roll production. The use of innovative fabrication processes and new materials entails the need to modify the existing construction of transponders, both: in relation to antenna circuit designs and matching systems as well as chip attaching methods. This task is difficult due to the lack of full information about the parameters of the functional inks and the process accuracy impact on properties of obtained electronic layers. In addition, materials for the ink-jet technology available on the commercial market are dedicated for applying on well-defined substrates (e.g., PET —polyethylene terephthalate, PI—polyimide) using technical infrastructure in an exact manner recommended by manufacturers—for example, an attempt to print on an uncommon substrate (e.g., paper) involves the necessity of carrying out many preliminary tests in order to modify the material surface or the ink composition. This causes the need to examine the developed designs in terms of material and process compatibility, especially when non-standard laboratory equipment is used—for example, the results of ink-jet printing are strongly dependent on selected printer settings and environmental conditions in which it is carried out.

In this context, problems encountered when manufacturing flexible RFID tags dedicated to work in the UHF and HF bands are presented in [35,72]. If designers are aware of technology difficulties that they may face in the fabrication process, they can significantly accelerate the synthesis of the devices. 

Application of conductive inks on elastic substrates has to be carried out at a temperature lower than that allowed for plastics (usually below 250 °C) which effectively does not allow one to obtain high path conductivity (e.g., about 50 mΩ/□ for the NPS-J Harima). This significantly but negatively influences the effectiveness of RFID antennas because their resistance should be as low as possible [73]. In order to improve this parameter research are conducted towards adjusting printer setup parameters (regarding the temperature of the ink and substrate, the positioner speed, number of piezoelectric nozzles), shaping the ink stream (regarding drop volume and frequency of drops by changing parameters of electrical pulse that stimulate piezoelectric nozzles, i.e. duration, rate of slopes rise and fall, amplitude), modification and stabilization of technological process conditions (mainly by adjusting environmental temperature and humidity or by introducing additional processes such as laser conditioning of prints), etc. It should be also noted that the quality of obtained electric paths and their durability is affected by the appropriate pre-conditioning of the substrate materials (by washing, drying, plasma treatment, etc.). An additional difficulty is the need to predict the degree of scaling the pattern in relation to the expected printout due to the real width of paths created by ink-jet. The width of electric paths is dependent on rheological properties of the used ink, the substrate surface type and condition as well as the printing device settings (for example the paths of HF antenna pattern had to be narrowed in the design by 160 μm and the UHF antenna pattern—by 80 μm [66,70]). The very important problem is also with adhesion of printed paths to the substrate—the effectiveness of the developed technology can be checked in this respect by the standard ASTM D3359-17 procedure [74]. 

**Figure 9:**
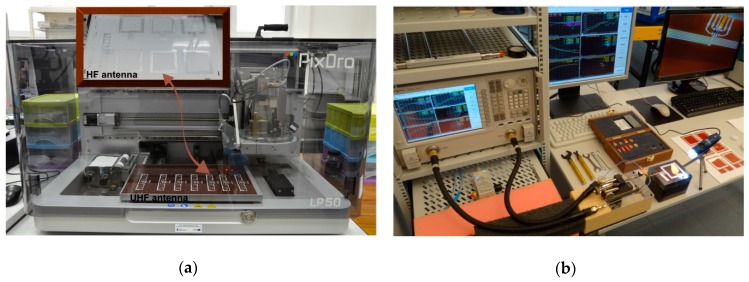
The ink-jet printed flexible antenna: (**a**) UHF and HF tag antennas under manufacturing process; (**b**) HF tag antenna under tests.

Taking into account the multitude of factors affecting the results of the technological process, it is obvious how important is to develop own know-how in the research laboratory. It should be emphasized that in order to develop a robust RF system, it is necessary to precisely identify not only the influence of the processes’ accuracy but also the parameters of new materials used [29] and to skillfully apply this knowledge at the design stage [32,34,35]. The worked out procedural knowledge was applied to the project of antenna dedicated to the UHF AMS SL900A chip (Figure 9a) [72]. The correctness of obtained design can be confirmed by convergence of measured and calculated impedance values of inductive circuit (Table 2). This parameter has to be adjusted (after taking into account the matching circuit) to the impedance of the RFID chip input circuits (Section 8) [75,76]. It can be found in Table 2 that there is relatively good convergence (regarding the real and imaginary parts) between values of measurements for subsequent test antennas S.#1–S.#7. It also indicates that the technological process is repeatability. However, increased values of resistance and reactance noted with reference to the model calculations result from the identified defects in the path printouts and designed model mapping inaccuracies.

An example of an antenna operating in the HF band and dedicated to the STM M24LR16E-R chip [77] (Figure 9b) is presented in [35]. Also, in this case, the main determinant of the correct implementation is the convergence of the measured and simulated impedance values. The inductance of antenna loops should be adjusted to chip input capacity in order to establish resonant circuit at the frequency *f*_0_ (Section 8) [75,76]. Based on the obtained results, it is possible to conclude that the measurements for subsequent test samples are very consistent and values are slightly higher than those obtained from the numerical calculations for the prepared model (Table 3). The very good convergence between the measured and simulated results is obtained when typical PCB technology and standard FR-4 substrate are used (Figure 5, Table 4), what predispose this method to fabricate tests samples in research experiments on RFID sensors [32].

In all cases, the antenna designs were elaborated in the Mentor Graphics HyperLynx 3D EM (HL3DEM) software environment. The LP50 inkjet printer (PixDro, Meyer Burger Ltd, Gwatt (Thun), Switzerland) was used for fabricating antennas (Figure 9a). The conductive layers were printed on Kapton HN-500 substrate (DuPont, Wilmington, DE, USA) using NPS-J ink (Harima Chemicals, Tokyo, Japan) with nanosilver particles. The impedance of tested antennas was determined on the basis of *S* parameters measured using a two-port vector network analyzer (VNA Keysight PNA-X N5242A) [78].

## 6. Determination of Material Parameters

The accurate design of high-frequency devices is only possible when the physical parameters of the construction materials are well known [79]. In the case of RFID transponders, the essential components that have the most important impact on proper operation of tags are the substrate and the housing (protective layer) that protects against the influence of environmental conditions. Usually they are made of dielectric materials which are characterized by the relative permittivity *ε*_r_ (in manufacturers’ catalogs defined as Dielectric Constant—DC) and loss tangent tg*δ* (Dissipation Factor—DF). Usually, constant values of these parameters are specified in manufacturers’ notes in a defined frequency range or for several selected frequencies (as a rule they are determined for the fundamental frequency and its harmonics, which further increases inaccuracy when reading parameter values by approximating measurements for the given point of curves tg*δ*(*f*) or *ε*_r_ (*f*)). It should not be surprising because these parameters are usually indicated according to the needs of the largest group of potential industrial recipients. Conducting research in the RFID technology, designers have to cope with this problem and have to designate these parameters alone. 

In this respect, the best description of the desired parameters can be found for the materials used in RF systems, in particular for copper-clad microwave laminates and ceramic substrates (e.g., Al_2_O_3_ or "green tape" dedicated to LTCC technology). Nevertheless, the latest material technology products, including specially prepared paper substrates [80], various types of composites [81], flexible plastics [82], fabrics [83], etc., are also used to construct RFID transponders. It should be noted that the same material compositions or similar or even the same names do not always mean a product with the same properties. For example, dielectric permittivity for the FR4 material that is commonly used in the production of PCB electronic circuits, not only changes with the frequency increase but it also depends on the lattice of glass fibers and resin content [84] and thus the technology used by a manufacturer.

The IPC-TM-650—Test Method Manual, Section 2.5—Electrical Test Methods [85] procedures proposed by IPC (the Association of Connecting Electronics Industries) can be used to determine the materials dielectric parameters. They are dedicated to specific frequency bands (e.g., 2.5.5.3—several hundred MHz, 2.5.5.9—1.5 GHz, 2.5.5.5—above 3 GHz) and impose some limitations on the tested material samples (e.g., 2.5.5.3—measurements of thin substrates, up to about 6.5 mm, but in the low UHF band; 2.5.5.9—not dedicated to materials with low dielectric losses, which are used in RF devices; 2.5.5.5—dedicated to measurements at frequencies above the UHF band). In addition, they require using special test stands that are rarely available in research laboratories due to the high costs. Professional project teams in RF technology more often have expensive but universal apparatus (e.g., two-port vector network analyzer) and appropriate software tools. In this case, more accurate results regarding determination of the dielectric parameters can be obtained on the basis of experience and the theoretical knowledge on microwave resonators. 

**Figure 10:**
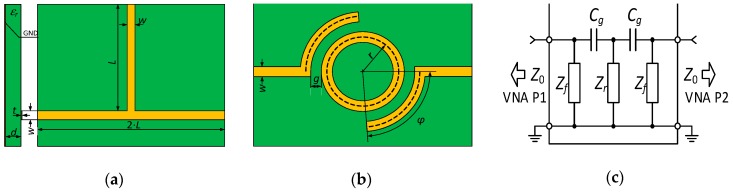
Microstrip resonator models: (**a**) T-resonator; (**b**) Modified ring resonator; (**c**) Equivalent circuit of modified ring resonator.

The problems related to determination of dielectric parameters for any frequency in the UHF band (in the range from 300 MHz to 3 GHz) is presented in [29]. The T-resonator and modified version of the ring resonator, presented in the Figure 10a,b, are used in the studies. The constructions dedicated to specified frequencies are developed in order to adjust the resonator impedance, described by the dependence (1), to the value of 50 Ω (i.e. input impedance of vector network analyzer). Appropriate calculation procedures based on the equations yielded from the analysis of resonator microstrips is proposed for this purpose [29,86].

Assuming *w* > *d* in the experimental work (computational procedures can be also adapted for rare cases where *w* < *d* or *w* = *d*), the input impedance *Z_f_* of the designed structures can be presented in the form:

(1) $$Z_{f}=\frac{120\pi }{\sqrt{\varepsilon_{e}}\cdot \left(1.393+\frac{w_{e}}{d}+\frac{2}{3}\ln\left(\frac{w_{e}}{d}+1.444\right)\right)},$$

where the effective dielectric constant is defined as:

(2) $$\varepsilon_{e}=\frac{\varepsilon_{r}+1}{2}+\frac{\varepsilon_{r}-1}{2}\cdot {\left(1+12\cdot \frac{d}{w_{e}}\right)}^{-0.5},$$

and effective width of the microstrip structure is:

(3) $$w_{e}=w+\frac{t}{\pi }\left(1+\ln\left(\frac{2\cdot d}{t}\right)\right),$$

and the other dimensions are described in the models in Figure 10a,b. The basic resonant frequency of the structure depends on the tuning element length *L* (in the case of the T-resonator) or the inner loop radius *r* (for the modified ring resonator) according to the following relationships:

(4) $$L=\frac{nc}{4f_{0}\sqrt{\varepsilon_{e}}},$$

(5) $$r=\frac{c}{2\pi f_{0}\sqrt{\varepsilon_{e}}},$$

In the case of the modified ring resonator, the angle describing the length of the coupling segment between the main ring and the supply paths:

(6) $$\varphi =\frac{\lambda }{4r},\;\mathrm{rad},$$

as well as the total impedance *Z* of all components (Figure 10c) have to be also determined.

**Figure 11:**
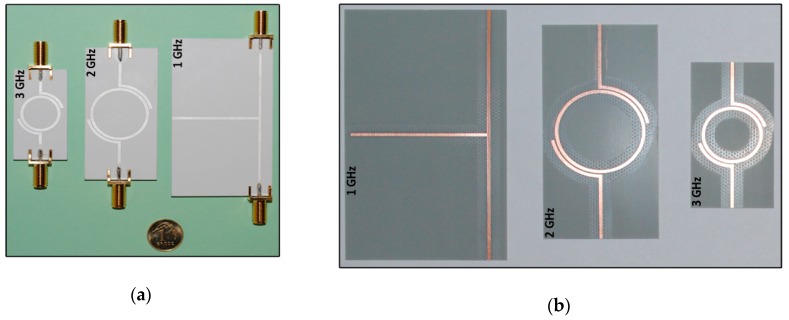
Test resonators: (**a**) On LTCC substrate; (**b**) On PCB substrate.

**Figure 12:**
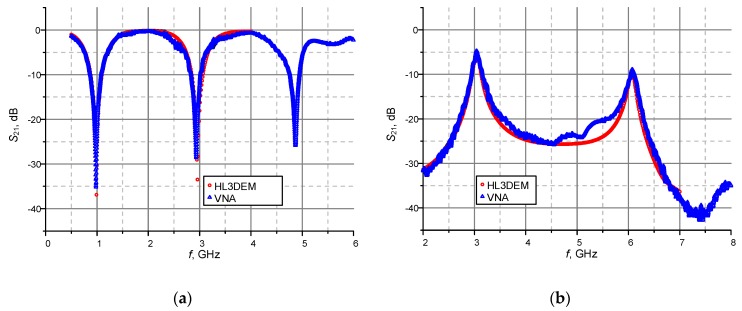
Confirmation of measurement procedures: (**a**) For T-resonator, LTCC FERRO A6-5 substrate, *f*_0_ = 1 GHz; (**b**) For modified ring resonator, ISOLA FR408 substrate, *f*_0_ = 3 GHz.

Based on the dependencies (1)–(6) and measurements of prepared resonators (Figure 11), the dielectric parameters of the substrates used in research projects can be determined. The tests were performed with materials dedicated to RF printed circuit boards: ISOLA FR408 and those used in LTCC technology: FERRO A6-S (Figure 12). The obtained results are verified on the basis of numerical calculations conducted in Mentor Graphics HyperLynx 3D EM (HL3DEM) software and experimental determination of *S*_21_-parameter by using the VNA (Keysight PNA-X N5242A). The convergence of the curves presented in Figure 12 confirms correctness of the elaborated procedure.

## 7. Synthesis of Antennas for RFID Sensors

The requirements for synthesis of antennas dedicated to RFID sensors (semi-passive RFID transponders with increased load capacity of energy harvester) operating in the UHF band are discussed in [34], whereas the problems related to the design of solutions dedicated to work in the inductive coupling conditions (the HF band) is brought up in [32] and [35]. In both cases, the special attention is paid to design inductive circuits for semi-passive transponders equipped with an energy harvester, with the possibility of providing power to electronic circuits dedicated to performing additional functions. The problems of designing and matching the antenna and chip impedance has to be considered differently for the HF and UHF bands, and moreover constructions and standard calculation procedures known from typical 50 Ω or 75 Ω signal paths cannot be used. 

**Figure 13:**
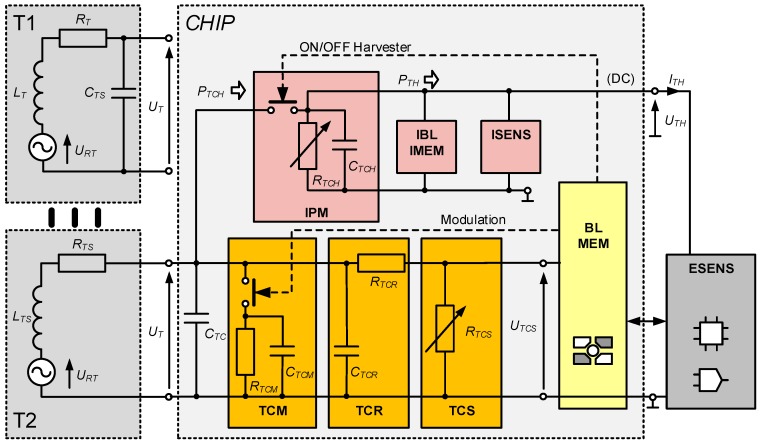
Block diagram of the modified model of semi-passive RFID transponder-sensor (block designation according to Figure 3). T1—parallel model of antenna; T2—corresponding serial model of antenna at given frequency; *L_T_*—self-inductance of antenna loop; *R_T_*—resistance of tracks and heat losses in antenna loop; *C_TS_*—resultant of all inter-turn capacities in antenna; *R_TS_* and *L_TS_*—resistance and inductance in serial antenna circuit antenna at given frequency; *U_TH_* and *I_TH_*—constant voltage and current at output of energy harvester; *U_RT_*—voltage induced in antenna loop; *P_TCH_/P_TH_*—input/output power of energy harvester; *R_TCR_*, *R_TCS_*, *R_TCM_*—resistance of respectively: voltage rectifier, voltage regulator and modulator; *C_TCM_*—capacity of modulator; *C_TC_*—input capacity of RFID chip.

The most important feature of RFID transponder antennas is that their impedance *Z_TA_* has to be expressed by a complex number that should correspond to the complex impedance value *Z_TC_* measured at the RFID chip terminals [76] and cannot be described by the real value 50 Ω or 75 Ω as it is in the classical radio systems. According to the equivalent serial circuit (presented in Figure 13) the antenna impedance has an inductive character and can be described by dependence:

(7) $$Z_{TA}=R_{TS}+jX_{TS}=R_{TS}+j\omega L_{TS},$$

where *R_TS_* and *X_TS_* are resistance and reactance of antenna, respectively, *L_TS_* is serial inductance and *ω* = 2π*f*_0_ describes pulsation.

On the contrary, the chip impedance *Z_TC_* has a capacitive nature and is strongly influenced by strength of the electromagnetic field. The parameter *Z_TC_* is expressed as:

(8) $$Z_{TC}=R_{TC}+jX_{TC}=R_{TC}+\frac{1}{j\omega C_{TC}},$$

where *R_TC_* and *X_TC_* are resistance and reactance of chip input circuits, respectively, *C_TC_* is capacitance and *ω* = 2π*f*_0_ describes pulsation.

In antenna of inductively coupled (the HF but also LF bands) RFID systems, it is important to achieve resonance in a parallel circuit of the inductance *L_TS_* and the chip internal capacitance composed of parallel connected components: *C_TC_*, *C_TCR_*, *C_TCH_*, where value *C_TC_* dominates significantly (Figure 13). In the HF band (*f*_0_ = 13.56 MHz), the antenna circuit is implemented in the form of loops with dimensions much smaller than the wavelength (λ ≈ 22 m). The highest value of voltage *U_T_* generated at the terminals is obtained when there is a parallel resonance between the inductance *L_TS_* of the antenna substitute circuit and the capacitance *C_TC_* of the active chip’s input that expresses capacitive character of the power supply. When the resonance is established there are the best conditions for obtaining energy from the signal generated by the RWD. The boundaries of the interrogation zone are described by minimum magnetic field strength *H_min_* at which correct data transmission between the RWD and tags can take place. In the case of energy excess, its part can be provided to the additional power supply block (IPM), then accumulated (e.g., in a supercapacitor) and used for additional tasks (e.g., measurement data acquisition). 

According to the method of designing effective RFID system operating in the UHF band presented in [58], the *Z_TA_* = Z_TC_* relationship should be satisfied at *P_Tmin_* [76]. This condition indicates the impedance matching between the antenna and chip input circuits (the *X_TA_* antenna reactance has inductive character and *X_TC_* chip—capacitive). Then, the power transmission ratio is expressed by the dependence:

(9) τ=4Re(ZTA)Re(ZTC)Re(ZTA+ZTC)2+Im(ZTA+ZTC)2,

and is equal *τ* = 1 which ensures maximum efficiency of energy transfer. The boundaries of the interrogation zone are described by minimum power *P_Tmin_* at which correct data transmission between the RWD and tags can take place.

The electronic chip is designed to be supplied by the minimal voltage *U_Tmin_* (HF band) or the minimal power *P_Tmin_* (chip sensitivity in the UHF band) which are enough for activating internal circuits of the transponder that is located at the interrogation zone. If the power load of the internal supply harvester increases by the current of execution blocks for additional functions, the minimum values of these parameters also increase, thus the IZ decrease. 

## 8. Determination of RFID Chip Parameters

Looking through the literature (both scientific as well as technical specifications provided by manufacturers), it can be noticed that there are many shortcomings and inaccuracies in the definition and determination of RFID device parameters. This observation refers particularly to transponder constructions dedicated to the UHF band, supplemented with additional functions which could be used in research on autonomous RFID sensors. This is due to the fact that some phenomena occurring in this type of radio systems have to be considered differently than it is assumed in the classical antenna theory [74,78], otherwise measurements are false. Therefore, designers of RFID systems have to elaborate their own know-how that could allow them to independently estimate the data needed to carry out the effective synthesis of RF devices. Determination of the RFID chip impedance *Z_TC_* as well as the atypical measuring stand for determining this quantity and the chip sensitivity *P_Tmin_* or minimum magnetic field strength *H_min_*, together with a discussion of the principles underlying operation of the RFID systems are presented in [76,87,88]. On the basis of worked out results, it is possible to determine the read range (maximal distance between centers of the tags and RWD antenna) as the main parameter of the IZ that describe efficiency of every RFID system. 

The problem concerns especially the impedance *Z_TC_* in the UHF band that changes along with fluctuations of the *P_T_* power level which is gathered from the unstable electromagnetic field in the input circuits (rectifier and voltage regulator, Figure 13) of the chip [89]. Therefore, it depends on a location and orientation of the transponder in relation to the RWD antenna as well as on parameter settings in communication protocols (e.g., ISO/IEC18000-63). In addition, the load of energy harvester generated by the digital system BL is not also constant as it depends on the currently performed function in the communication process (i.e. writing is more energy consuming than reading from the internal data memory). In the case of RFID sensors, a current operation performed by the data acquisition block (i.e. measurements, serial interface activation) furthermore significantly affects the energy balance of the electronic system. On the other hand, the accuracy of determining the *P_T_* parameter depends on the impedance matching in measuring path of the research bench (but the chip impedance is strongly dependent on the *P_T_*). The best impedance matching between the chip and connected antenna is obtained when the power transfer coefficient τ is equal 1 (9) and then all experimental measurements should be performed.

**Figure 14:**
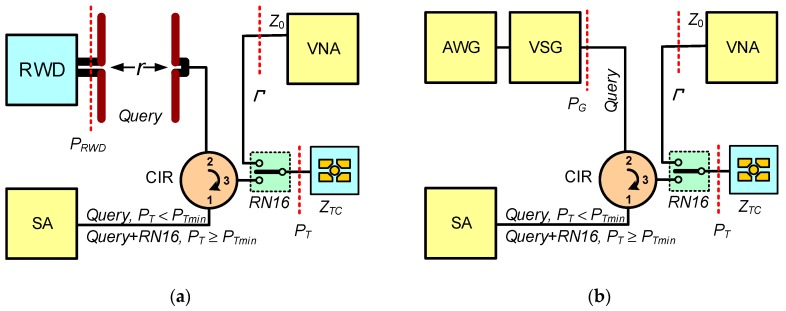
Methods of chip parameter determination: (**a**) Means by using RWD; (**b**) Means by using signal generator. SA—signal analyzer; VNA—vector network analyzer; AWG—arbitrary wave generator; VSG—vector signal generator; CIR—circulator.

Therefore, special test procedures to indicate the chip sensitivity that is necessary to determine the chip impedance have to be applied. The two methods (Figure 14) based on looking for the minimum power *P_Tmin_*, at which the transponder responds to the *Query* call (according to the communications protocol ISO/IEC 18000-63: *Inventory Order*) from the RWD, sending back the *RN16* string (according to the communications protocol ISO/IEC 18000-63: *16b Random or Pseudo-Random Number*) are proposed in [76]. This answer is generated only when the energy conditions for powering chip (*P_T_* ≥ *P_Tmin_*) are fulfilled. Due to the use of a ferrite circulator, it is possible to append a vector network analyzer that allows to observe the transmitted signals and, primarily, to determine the actual power *P_Tmin_* (assuming that losses of test cables are known). When determining the power value, the mismatch of the chip impedance (*Z_TC_*) and 50 Ω of the measuring path (*Z*_0_) have to be also taken into account:

(10) $$P_{Tmin}=P_{min}\left(1-{\left|\Gamma \right|}^{2}\right),$$

where *Γ* is the reflection coefficient measured by the vector network analyzer at the output power *P_Tmin_* and at the input gate plane shifted to the point of chip and its antenna connections (connection pads) [90].

The main difference between the developed methods results from the way of simulating the real communication link. In the case of using a long range read/write device (with the possibility of interfering in the communications protocol and adjusting level of the output power *P_RWD_*), application conditions of given RFID system can be reflected precisely (Figure 14a). Such a setup of the laboratory bench is particularly useful when development studies are conducted according to a real implementation of the automatic identification. It allows to test the settings of the target communication path (e.g., RWD device output power *P_RWD_*) or the impact of environmental disturbances (e.g., metal objects proximity, obstacles affecting the electromagnetic field). In the second proposal (Figure 14b), the communication path is simulated using two generators: of vector and arbitrary signals. Patterns of the transmission frame are built using software procedures prepared in the Mathcad software according to protocols described in the relevant standards, so that it can be subjected to any modifications. It is also possible to accurately determine the output power *P_G_* for the carrier obtained from the vector generator which is routed to the input of the tested RFID chip—then the influence of environmental conditions is omitted. An important disadvantage of this method is the very high costs of control and measurement equipment used to simulate operation of the RWD device.

**Figure 15:**
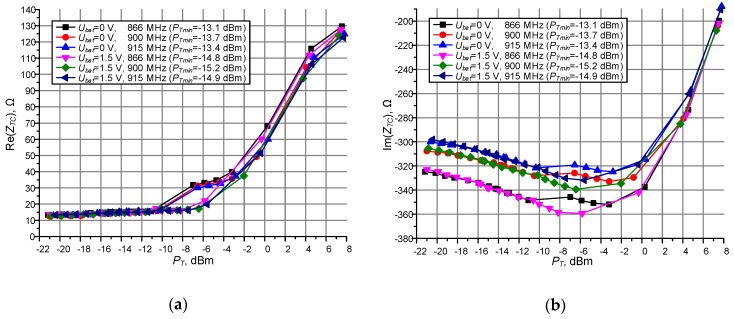
Examples of research results: (**a**) Real part of chip impedance *Z_TC_*; (**b**) Imaginary part of chip impedance *Z_TC_*. *P_T_*—power transferred to chip; *P_Tmin_*—chip sensitivity.

The proposed procedures are verified on the basis of experimental tests with RFID chip AMS SL900A. The obtained results (Figure 15 and Table 5) are convergent for the both methods at common settings assumed in the tested system and correspond to data specified in manufacturers’ catalogs. It should be noted that the chip sensitivity and its impedance are affected by a wide variety of factors (i.e. operating frequency, protocol parameters, chip operation mode, performing of additional functions in RFID sensor). It is usually not presented in the catalogs but has to be included in the synthesis of RFID devices, in particular when determining the interrogation zone. The small variations of the sensitivity which are observed in the obtained results have a noticeable influence on the read range *r_Max_* and thus the IZ of RFID systems (Table 6) and can be important when selecting the appropriate RFID equipment for a given implementation.

Yet another conclusion can be drawn from the presented results of the semi-passive chip impedance measurements. The value of the real and imaginary part measured at the *P_Tmin_* power is not influenced by using the battery additional source. Therefore, the same antenna constructions can be used for both passive and semi-passive modes.

## 9. Determination of RFID Antenna Parameters

Determination of the antenna parameters in a precise way is the key task both in verification of the design process of efficiently working RFID sensors as well as in definition of the interrogation zone in a real automated identification system. The problem is complicated by the fact that there is not universal pattern of the inductive circuit that could be implemented in any application. In fact, all components of a RFID system have to be individually designed, appropriately to the established assumptions and expected environmental conditions. The systematic characteristic of the problems connected with measurements of antenna parameters that are applied in the RFID technology are presented in [78] and [91]. The indirect method for determining the impedance is also proposed in these works as well as the obtained results of conducted research are analyzed and concluded.

**Figure 16:**
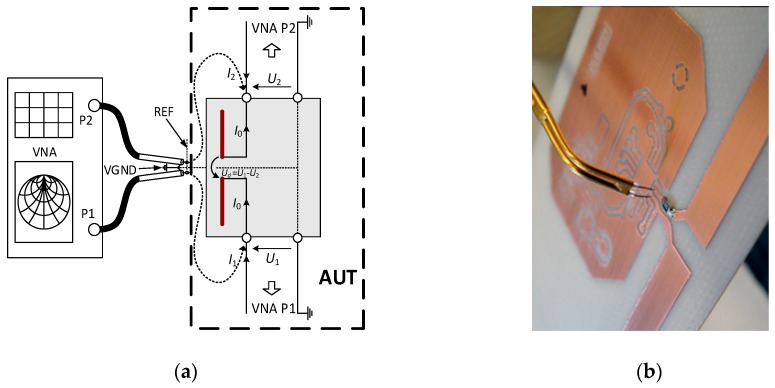
Determination of impedance in RFID antenna: (**a**) Idea of method; (**b**) Attaching probes to antenna under tests.

The value of the *Z_TA_* impedance in the LF band can be easily determined using a typical RLC bridge. In other cases, it is necessary to apply the special methods in which a vector network analyzer equipped with two unbalanced ports (50 Ω) and a passive differential probe PDP (Figure 16a) are essential equipment of the test stand. The procedure consists in measuring the scattering matrix and estimating the differential impedance on the basis of derived dependence [78]:

(11) $$Z_{T}=2Z_{0}\frac{S_{12}^{2}-S_{11}^{2}-2S_{12}+1}{{\left(1-S_{11}\right)}^{2}-S_{12}^{2}},$$

assuming symmetry of the measurement channels P1 and P2 of the vector network analyzer: *S*_11_ = *S*_22_, *S*_12_ = *S*_21_ and *Z*_0_ = 50 Ω.

**Figure 17:**
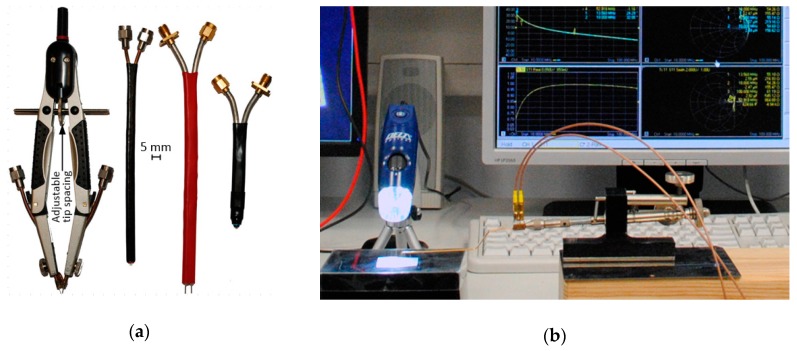
Equipment of measurement stand: (**a**) Handmade probes; (**b**) Professional equipment—precise probes and micromanipulator.

In order to conduct experiments precisely at the laboratory it is necessary to prepare a measurement stand with special differential probes adapted to various shapes and sizes of tested components (Figure 16b). The tips of the probes can be manufactured by hand with very high accuracy (Figure 17a) especially that raster of connection terminals is not standardized. In order to ensure good contact with delicate and tiny pads of the antenna or chip it is better to use dedicated professional probes (Figure 17b) and precise micromanipulator (e.g., 44-8000-D-NA PDP). It should be noted that common RF connectors (e.g., N, SMA, UFL) cannot be applied due to technological and EMC compatibility aspects.

Evaluation of the presented method dedicated to determining the impedance of RFID device components was carried out on exemplary test antennas made in the PCB technology. It was made by comparing measurements with the numerical calculations obtained in the Mentor Graphics HyperLynx 3D EM program (HL3DEM) at the antenna circuits design stage. The measurements of *S* parameters were performed on the laboratory stand equipped with two port vector network analyzer (VNA Keysight PNA-X N5242A), micropositioner, manufactured differential probes, test cables and calibration elements. The parameters of used substrates (thickness, relative permittivity, loss tangent), for conductive layers (thickness, resistance) and assumed pattern of coils (number of loops, side length, distance between coils, track width) were provided for the calculations by measurements conducted in the author’s laboratories. All antenna versions were manufactured using the appropriate technological devices: CNC plotter (LPKF ProtoMat S100) and ink-jet printer (PixDro LP50).

**Figure 18:**
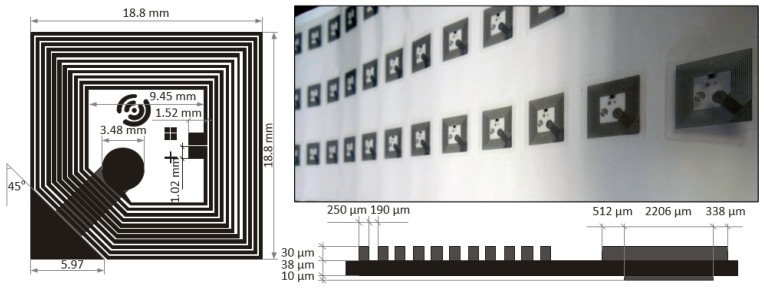
Test antenna of the HF band—parameter designation for the model of commercial antenna.

Examples of research results in the field of designing antenna for autonomous RFID sensors of the HF band (operated at increased load conditions generated by additional blocs/functions) are presented in [32,35]. Two different antenna designs are dedicated to STM M24LR16E-R chip. In the first case, a typical PCB technology and standard FR-4 substrate (Figure 5) is used for fabricating samples [32] while in the second—the conductive layers are implemented by using method of printing Harima NPS-J inks with silver nanoparticles on DuPont Kapton HN-500 flexible substrate (Figure 9b) [35]. A very good convergence between the measured and simulated results is obtained (Table 4) in the case of the PCB antennas, as this technology is well known and easy to use on the prototype stage. The discrepancies obtained for subsequent test versions of flexible antennas (Table 3) result mainly from corrections introduced in the technological process settings aimed at increasing the accuracy of mapping developed project [35,72]. Both versions of antennas were tested in the RFID sensors (Figure 5) that were designed on the basis of microprocessor data acquisition system (with the C8051F988 microcontroller) which enabled to measure various physical quantities including those from the LTCC thick-film pressure sensor developed as a part of earlier research described in [92]. The similar works were carried out for the HF band antennas dedicated to the NXP NT2H1001 chip (Figure 18) [91].

**Figure 19:**
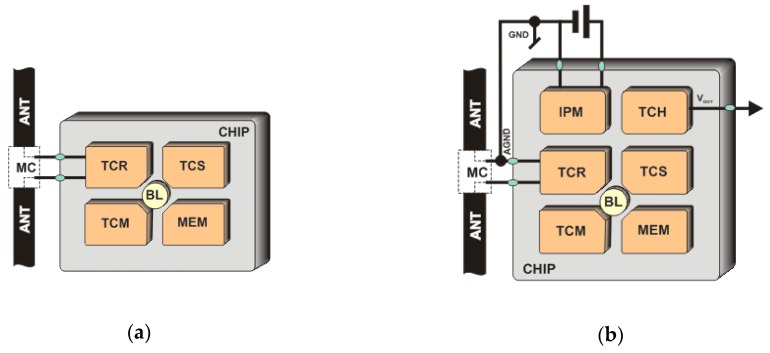
Connecting antenna to chip: (**a**) Passive construction; (**b**) Semi-passive construction with function of providing voltage output from internal energy harvester. GND—chip ground, AGND—antenna ground.

In the typical passive transponders of the UHF band, some basic methods are used to design high-reactance antennas [93], including modification of microstrip antenna shapes (by changing the coupling with construction materials of marked objects), application of T-type matching circuit, coupling loop etc. (Figure 19a). Unfortunately, they do not operate properly for semi-passive transponders (e.g., AMS SL900A) in which the antenna and chip are placed on the same ground in order to facilitate the implementation of a supplementary power source (Figure 19b). In such a situation, it is necessary to use symmetrical or asymmetrical antennas with open arms where the matching is made using microstrip structures or passive SMD components. 

**Figure 20:**
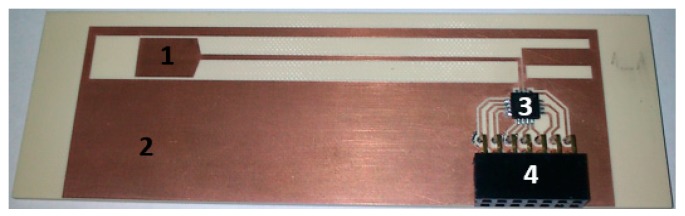
UHF antenna dedicated to AMS SL900A chip. 1—radiator; 2—ground; 3—chip; 4—connector.

The UHF antenna structure dedicated to cooperating with AMS SL900A and designated to use in proximity of metal surfaces, for implementing in practical application, is presented in Figure 20. This component can be designed properly only when the accurate values of dielectric parameters are known. Especially it concerns substrates that are used in structure of transponders but also information on materials from which product intended for identification is made. In the presented case, the IS-680-300 copper clad laminate (ISOLA GmbH, Düren, Germany) dedicated to fabrication of high frequency devices is used.

**Figure 21:**
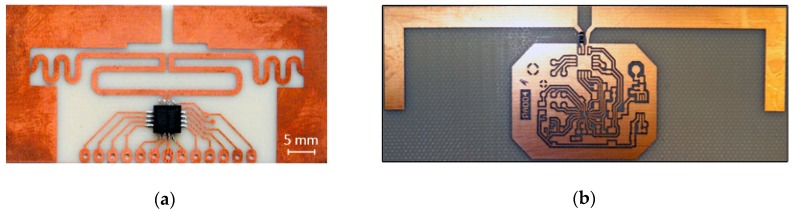
Test antennas of the UHF band: (**a**) Transponder designed to laboratory tests; (**b**) Transponder dedicated to work as the RFID sensor.

**Figure 22:**
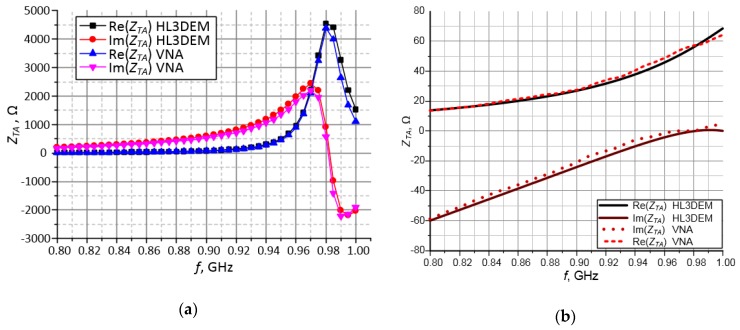
Results of experiments: (**a**) Transponder designed to laboratory tests—Figure 21a; (**b**) Transponder dedicated to work as the RFID sensor—Figure 21b.

Taking into account the specific requirements of target application, the antennas presented in Figure 21 were also designed for the use with a passive/semi-passive AMS SL900A chip. The satisfactory convergence is achieved in terms of practical usability. However, small differences are noticeable on the determined courses *Z_TA_*(*f*) (Figure 22). On the basis of the acquired data analysis, feedback information about the technological process accuracy as well as verification of numerical calculations, it can be indicated that visible discrepancies are caused by not sufficient resolution of a CNC machine on which the pattern of conductive paths is made. The fact that substrate dielectric parameters (relative permittivity and loss tangent) are assumed to be fixed in the frequency domain have also significant impact on the obtained results—this assumption of constant values is not consistent with reality and it is characterized in [29].

The antenna radiation pattern is another important parameter that has to be taken into account when transponders dedicated to work in the UHF band are designed [75]. In the case of typical constructions used in commonly known radiocommunications systems (DVB-T, GSM, UMTS, LTE, WiFi, etc.), the measurement methods known from the classical antenna theory can be applied [94]. However, considering RFID transponders, there is a problem associated with the previously mentioned impedance expressed in a complex number. The input impedance of the chip is constantly changing during its operation and depends on the electromagnetic field parameters in the considered point of space. It is also strongly influenced by the distance, orientation according to the RWD antenna, the marked object construction, the environmental conditions of radio waves propagation, etc. Known methods for determining the radiation pattern [95,96] can be found in the literature of the subject but a very advanced and expensive apparatus is used in them or a measurement process is very complicated. But, in publication [75], a modified method without the mentioned drawbacks is proposed. The measurement procedure is based on common RFID devices and standard equipment of the typical radio frequency laboratory as well as specially prepared software tools what is its great advantage.

It should be noted that the main problem to be solved is how to maintain the constant input impedance *Z_TC_* of the chip during the directional diagram determination, when changing polar coordinates *θ*, *φ* of the antenna under tests (AUT). The only point where this parameter is unambiguously defined is the boundary surface of the interrogation zone. It can be indicated by the minimum power *P_Tmin_* that is necessary to ensure the energy and communication conditions for the transponder operation [76]. Searching for *P_Tmin_* values is based on changing distance *r* between the antennas (AUT and RWD). Position changes of the antennas cause the need to use an advanced positioning system and to maintain the same environmental conditions in the whole measurement space. Therefore, a special procedure is proposed in [75] for determining this boundary condition by adjusting the power *P_RWD_* delivered to RWD antenna terminals. It is adapted to the equipment of a typical RFID laboratory, including primarily an anechoic chamber, digitally controlled positioners of AUT (at angles *θ*, *φ*) and RWD (polarization change) and a commercially available read/write device with an adjustable attenuator on the output. In order to automate the measurement process, control algorithms for the used devices is developed in the LabView program. To determine the standardized *F_Tn_* directional diagram, the basic relationships are used:

(12) $$P_{Tmin}=P_{RWDmin}\frac{G_{R}G_{T}\lambda^{2}\tau \chi }{{\left(4\pi r\right)}^{2}},$$

(13) $$F_{Tn\;dB}\left(\theta ,\phi \right)={\left[P_{RWDdBm}\left(\theta ,\phi \right)\right]}_{min}-P_{RWDdBm}\left(\theta ,\phi \right),$$

where *P_RWD_* means power supplied to RWD antenna terminals, *G_R_*—RWD antenna gain, *P_T_*—power received at transponder antenna, *G_T_*—antenna transponder gain, *χ*—polarization correlation coefficient of the radiocommunication antenna system, *τ*—power transfer coefficient from transponder antenna to chip, *λ*—wavelength, *r*—distance between antennas and *P_RWDdBm_* is power measured in dBm.

The laboratory stand allows to perform measurements in conditions that are close to a real application: the antenna can be placed on a marked object during the measurements that is especially important in the case of RFID transponders. In addition, the procedure is carried out without any contacts and cables that could affect the diagrams—no items have to be attached to the transponder. 

**Figure 23:**
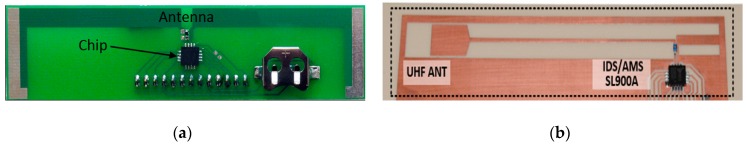
UHF RFID transponders used in experiments: (**a**) Project with omnidirectional antenna; (**b**) Project with directional antenna.

The proposed idea was tested on two exemplary UHF antennas (directional—Figure 23a and omnidirectional—Figure 23b that is a part of the demo boards of the autonomous RFID sensor presented in the Figure 2a) designed to operate with the AMS SL900A chip. 

**Figure 24:**
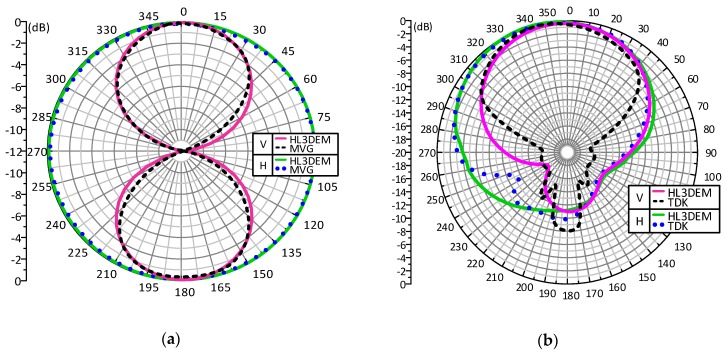
Confirmation of measurement procedure—comparison of measurement results with model diagrams for V polarization: (**a**) Project with omnidirectional antenna; (**b**) Project with directional antenna.

Directional diagrams of radiation patterns for V polarization are presented in Figure 24 for both test antennas from Figure 23, respectively. The high convergence of the measured and calculated results confirms the usefulness of the proposed method and minor discrepancies are caused by the simplifications assumed at the modeling stage (the infinite plane of the dielectric layer).

## 10. Determination of Interrogation Zones in Practical Implementation

The three-dimensional interrogation zone (IZ) is the most important parameter in the RFID technology that describes the effectiveness of an automated identification system [1]. This is the area around the RWD antenna in which communication and energy conditions necessary for the proper operation of RFID chips placed on marked products are fulfilled. The process of conducting radio-communication can be carried out with one (single RFID system) or with multiple tags (multiple RFID system) only when transponders connected with recognized objects are supply properly. 

The 3D IZ should be determined for the entire RFID application, which is not possible in many cases due to the lack of reliable information about the components. Specification of transponder parameters given in manufacturers’ catalogs is frequently incorrect for semi-passive structures with measuring functions. It is particularly difficult to simulate and design the multiple RFID systems where the interaction of variously distributed and oriented transponder antennas has to be additionally considered. Therefore, a time-consuming and uncertain trial and error method is more often used in the common practice in order to develop new RFID technology implementations.

The problem of determining the IZ area is outlined in [32] using the example of RFID systems that operate in the HF band. In the case of such applications, data exchange and energy transfer processes are accomplished by obtaining the inductive coupling (characterized by mutual inductance *M* and inhomogeneous magnetic field intensity *H*) between antenna of read/write device and tags. The transponder model (Figure 13) should be slightly improved when the sensor functions are considered. The load generated by an additional energy acquisition system has to be included in the synthesis process. Then, it is possible to determine the induced voltage value at the antenna circuit terminals (RFID chip input terminals) at a total given load as well as magnetic field parameters at the point where the transponder is located. The IZ area boundaries can be determined by searching for places where the condition of minimum magnetic field strength *H_min_* (14) is satisfied and thus correct level of energy is supplied to RFID chips and consequently the transponders response to the call from the RWD:

(14) $$H_{min}=\frac{\left|U_{Tmin}\right|}{\mu_{0}}\cdot \left|\frac{\left[1+\left(\frac{1}{R_{TCH}}+\frac{1}{R_{TCR}+R_{TCS}}+j\omega C_{T}\right)\cdot \left(j\omega L_{TS}+R_{TS}\right)\right]}{j\omega \cdot N_{T}\cdot S_{T}}\right|,$$

where *μ*_0_ = 4π⋅10^−7^ H/m and:

(15) $$U_{T}=U_{TCS}\cdot \left(1+\frac{R_{TCR}}{R_{TCS}}\right),$$

(16) $$U_{T}=\frac{j\omega \cdot M\cdot I_{R}}{1+\left(\frac{1}{R_{TCH}}+\frac{1}{R_{TCR}+R_{TCS}}+j\omega C_{T}\right)\left(j\omega L_{TS}+R_{TS}\right)}.$$

The verification of the developed model can be carried out on a specially prepared test stand where the IZ area is determined by searching for the maximum distance from the RWD antenna (*r_Max_*) designated by the respond of the transponders to the call from the read/write device (Table 7). The maximum distance is searched only in the axis of antenna system. The minimal value *H_min_* of the magnetic field intensity is measured using the R&S FSL18 spectrum analyzer and the HZ-14 near field probe but only for control purposes. Various load conditions of the RFID chip output on which the energy is providing from the internal harvester to external circuitry can be also simulated. The *P_TH_* power is determined based on measurements by a Tektronix DPO71254B oscilloscope equipped with the P7504 CT1 probe. The obtained results of *H_min_* measurements can be verified on the basis of the numerical model of RWD-transponder antenna system which was developed in [97].

**Figure 25:**
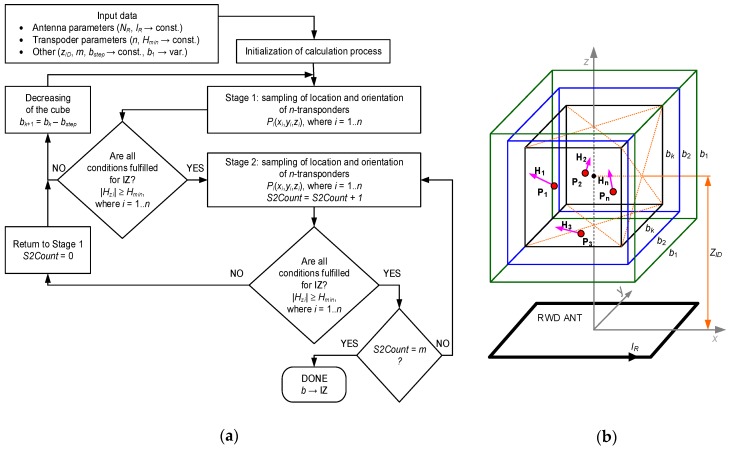
Procedure of 3D IZ determination: (**a**) Algorithm; (**b**) model of IZ.

The spatial arrangement of multiple transponders in the application operation area (called **Ω_ID_**) is analyzed in [33]. Investigations in this field described in the literature focus mainly on increasing the distance between centers of antennas in a typical arrangement: RWD antenna and the antenna of a one transponder [98] and analytic considerations concern simple geometric configurations and generally in its symmetry axis. However, in [33] the possibility of changing localization of multitude transponders placed in points *P*_1_(*x*_1_; *y*_1_; *z*_1_) ÷ *P_n_*(*x_n_*; *y_n_*; *z_n_*) and their orientation in the three-dimensional area **Ω_ID_** is implemented in the proposed model. Due to the fact that there is a highly inhomogeneous magnetic field in the IZ, the RFID chips are properly powered, and thereby they can generate a response to a call from the read/write device, when the condition *H_z_* ≥ *H_min_* (*H_z_*—magnetic field strength component in the *z* axis, perpendicular to the RWD antenna plane) is fulfilled at the given location (Figure 25b). 

In order to perform the analysis for each of the *n* transponders placed randomly in selected points *P_n_*(*x_n_*; *y_n_*; *z_n_*) of **Ω_ID_**, the Monte Carlo method [99] is used. This technique is quite often applied to solve research problems in the RFID technology, for example in the field of object localization [100], optimization of communication protocols [101], signal detection, collision elimination in multiple systems [102], etc.

**Figure 26:**
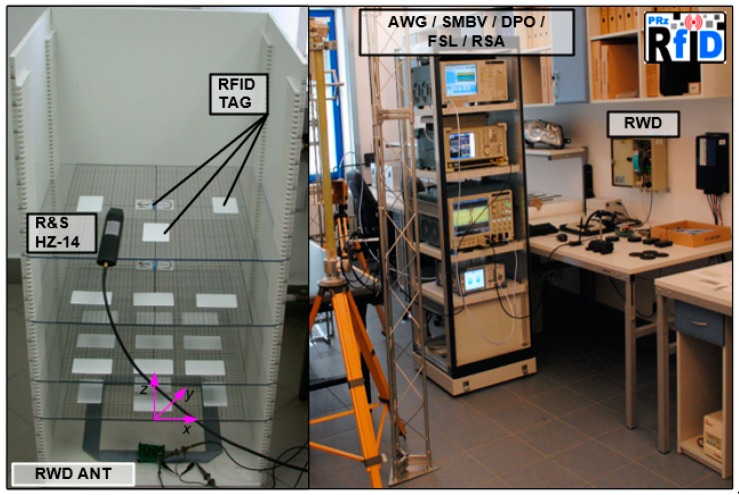
Laboratory test stand for determining 3D IZ. R&S HZ-14—near field probe; AWG—arbitrary wave generator AWG5002B; SMBV—signal vector generator R&S SMBV100A; DPO—oscilloscope Tektronix DPO71254B; FSL—spectrum analyzer R&S FSL18; RSA—spectrum analyzer Tektronix RSA 3408B.

The algorithm of the developed method is shown in Figure 25a. The input data are specified by testing equipment available in the authors’ laboratory (Figure 26). The IZ is approximated in the process of modeling with a cube placed centrally in relation to the RWD antenna (Figure 25b), at *r_ID_* distance from its center (relative to the cube center). The cube base is parallel to the RWD antenna plane, and in the next iterative steps its side length *b* is changed. The assumed group of *n* transponders is randomly arranged and the conditions necessary for supplying RFID chips and for generating response to the call command are verified. The effectiveness of identification *η_ID_* is determined on the basis of the obtained results:

(17) $$\eta_{ID}=\frac{l_{ID\_OK}}{n}\cdot 100\%,$$

where *l_ID_OK_* means the number of identified transponders.

Achieving the efficiency *η_ID_* = 100% for one selected arrangement does not guarantee the effective operation of the RFID system. Therefore, it seems necessary to consider all other cases of the *n* transponder allocation, which is impossible in practice. Thus, only by applying the large number law [99], the probability of determining the IZ boundaries can be defined.

To confirm effectiveness of the developed procedure for determining the IZ boundaries, the special test stand is prepared (Figure 26). The results of the cube sizes obtained in the conducted experiments have a large convergence with the numerical calculations (Table 8). It should be stated that in order to determine the interrogation zone, it is necessary to have precise information about parameters of all RFID devices used to build the implementation of automatic object identification system. A similar procedure can be performed for UHF systems, based on the search for points at which minimum power *P_Tmin_* is supplied to tags.

## 11. RFID Technology Implementation—Textronics

The above described research activity in the field of the RFID technology are reflected in developing new constructions e.g., such as the invention protected by patent PL 231291 B1 issued by the Polish Patent Office [103]. Its subject is a unique, in terms of design, electronic tag intended for use in textronic objects (marked with the abbreviation RFIDtex), during monitoring and automation processes at every stage of their life cycle (i.e. during production, storage, distribution, operation, maintaining service and utilization, etc.). RFIDtex is a textile product (e.g., general-purpose textiles, hospital linen, workwear and protective clothing, textiles for the hotel and catering sector—the HoReCa industry—as well as specialist clothing and other various purposes textiles) with which electrical and/or electronic components are permanently integrated in order to obtain intelligent construction with specific properties and functionalities. It is designed to be used used throughout entire life cycle of the textile products to support identification systems.

Typical RFID transponders are generally a combination of a chip and an antenna. The chip is permanently attached (through soldering, gluing, etc.) to the pads of the antenna by soldering or using different kinds of adhesives. The antenna is made as metal conductors applied on a substrate from material such as glass-reinforced epoxy laminate, flexible plastic or paper, ceramic, etc. Their shape and structure are designed in the way that allows the easiest attachment (permanently or not) to a marked object. Solutions of modern transponders are not sufficiently adapted to label textile products over their entire life cycle, where adequate flexibility, comfort of use and resistance against difficult environmental conditions (washing, chemical cleaning or ironing) are required. However, there are constructions, so-called laundry transponders, but they are integrated with the products outside the production plant of textiles, at the stage of creating an automatic identification system.

**Figure 27:**
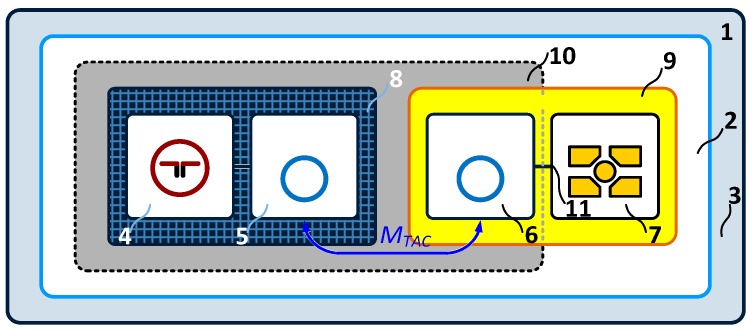
Idea of RFIDtex invention. 1—textronic product with RFID interface; 2—textronic structure; 3—material from which product is made; 4—stitched/embroidered antenna (but also: press-in, welded, glued, etc.); 5—antenna coupling system (made in the same technology as antenna); 6—chip coupling system; 7—RFID chip(s) of different frequency band; 8—sewn/embroidered antenna module made using techniques existing in the textile industry (by sewing, embroidering, ironing, gluing, welding, etc.); 9—microelectronic module (semi-finished product supplied to the textile industry); 10—antenna system; 11—point of attaching chip to antenna system; *M_TAC_*—mutual inductance of inductive coupling.

**Figure 28:**
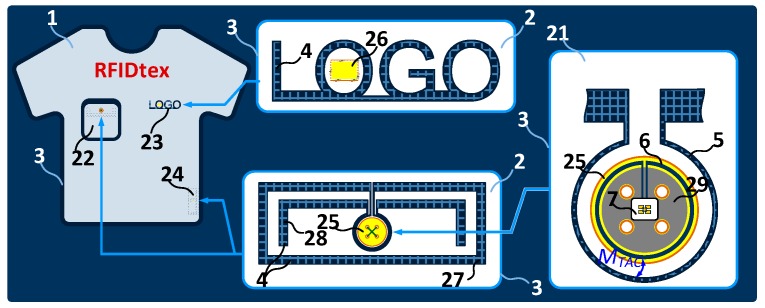
Implementations of textronic product with RFID interface. Implementations of textronic structure: 22—as hidden structure in internal layers of product, 23—in form of company logo, 24—in form of tag. Examples of microelectronic module implementation in the form of: 25—button, 26—emblem, strips/woven or other designs desirable in the textile industry. 21—part of textronic structure; 27—HF antenna; 28—UHF antenna; 29—magnetic, diamagnetic or ferromagnetic material; 1—7 as in Figure 27.

The use of coupling systems proposed in [103] allows separating the typical RFID transponder structure into two independently manufactured components (Figure 27). The antenna module can be embroidered with conductive threads, sewn or ironed into a textile material, and then the microelectronic module created in the form of an intermediate product (e.g., button or label)—can be easily integrated with a RFIDtex product. The main advantage of this solution is the flexibility and resistance gained against the impact of environmental conditions, with the possibility of using fabrication means typical for the textile industry. It reflects into a reduction of costs in managing production stage as well as in the comfort of creating object identification systems at the whole product life cycle (Figure 28).

The practical usefulness of the proposed textronic transponder consists primarily in the possibility of its use throughout the life cycle of a marked product, providing additional managing functions at every stage: production (e.g., improved quality control), storage (e.g., complete information about inventory levels), wholesale (e.g., dynamic recognition of products) and retail (e.g., immediate information about parameters, advertising) distribution, use (e.g., displaying to the intelligent devices the way of maintaining clothes), disposal (e.g., providing full information about the product material composition). The solution has a significant application in health care units where handling selected groups of textiles (e.g., hospital linen or protective cloths of surgeon) is regulated by strict laws that enforce to gather accurate information on the inventory (including, for example, its maintenance, conservation method, extent of the wear). Another purpose of the invention is the possibility of its use in home appliances (e.g., washing machines, vacuum cleaners, irons). For example, the correct process of washing the assortment thrown into the laundry machine drum can be automatically chosen and performed according to the information contained in the proposed textronic RFID transponder. Such a function may cause the invention to have the largest target group of users, i.e. all people who wear clothes. Similar examples of home appliances may concern automatic programming when ironing clothes as well as vacuuming, washing or cleaning carpets, upholstery and other general-purpose textile products.

The further benefits from applying the textronic RFID transponder appear when considering the latest achievements introduced into the RFID technology. For example, the implementation of constructions that operate in many frequency bands allows merging expectations of producers, distributors as well as consumers. The first group of the users gain benefits from enlargement of the read distance thanks to the possibility of applying long-range UHF RWDs. In turn, the individual consumers get access to intelligent functions of RFIDtex through an application installed on mobile devices, e.g., smartphones or tablets with Near Field Communication (NFC) options available only in the HF band (e.g., advertisements and specific data stored in worn or bought textronic clothing can be accessible on the mobile phone).

Considering the offer of the contemporary RFID market and technological possibilities of manufacturing radio-electronic devices, it should be noticed that the costs of implementing mutually incompatible identification systems and idea of their application are the main barrier to introduce the RFID transponders in the textile industry. At present, these costs have to be considered independently for each stage of the product life cycle. It is due to the lack of a uniform construction that could be appropriate for the production, logistics, use, disposal, etc. The proposed structure of the RFIDtex does not eliminate these expenses of implementing RFID system, but it constitutes the base for comprehensive service extended to the entire life cycle of the textile product. Since the RFID technology significantly improve logistic processes the additional costs of fabricating RFIDtex can be spread out at any stage, even if the special transponders need to be enhanced in order to meet specific requirements according to exposure to chemicals, high temperatures, etc. For example, if the transponders dedicated to cloths are fabricated in the production phase with a view of improving industrial processes and quality control, then they can be adapted to the further use of product in automated services such as laundry, product complaints, authorization, determination of durability and wear, protection against theft and even utilization.

## 12. RFID Technology Implementation—Localization and Navigation

RFID technology can be used to indicate the current position and to determine movement paths of a tracked object. It is feasible on the basis of reference network of RFID transponders, by identifying the nodes found in the interrogation zone. 

The Global Positioning System (GPS) is one of the most well-known location and navigation methods where the object position (e.g. person, car or rather a GPS device attached to an object) can be precisely determined in relation to the world map. Nevertheless, the radio signal sent by satellites is significantly attenuated by dense urban or industrial facilities and it is not available inside buildings. Therefore, other systems on the basis of which indoor localization processes could be carried out are the scope of investigations. The main research goes towards image analysis or implementations of various radiocommunications services, especially those standards that operate in the UHF band, i.e. wireless WiFi network [104], GSM radio transmission stations [105], FM [106], Bluetooth [107]. The accuracy of position determination in the mentioned radio systems is low, inherently limited to indication of a room where the object is located, in other words, to indicating localization of a base station (e.g., router), whereby the recognition zone is defined by router range. Attempts are also made to build more sophisticated solutions in which the power of signals sent by transmitters associated with objects and received by base stations are analyzed. Such systems allow to increase the accuracy to a few meters. The most important problem in this case is to predict and characterize the impact of environmental conditions on the transmitted electromagnetic waves. However, the most important advantages include, first of all, the possibility of using infrastructure that is installed to provide common wireless communications. There are also known research projects aimed at combining different methods of determining the objects localization and their support with additional measurement systems based, for example, on laser or ultrasonic transducers [108] as well as monitoring cameras [109]. Such solutions require advanced data processing implemented in digital devices with high computing power. 

The commonly used systems of security and access control, tracking items in industrial logistics (during parcels, materials or production item shipment), marking measurement samples or valuable materials in research processes (in various science, technology or medicine areas) should be primarily pointed out [110] in the aspect of the RFID technology applicability for localization and navigation of marked objects. A large part of the applications concerns Internet of things (IoT) [111], automatic identification of fast-moving consumer goods (FMCG) in global supply chains [112] or vehicle movements, e.g., in the rail or road transport area (Automatic Vehicle Identification or AVI) [113]. It should be noted that in all these implementations, the RFID transponders are attached to monitored objects that move through the operation area, and their localization is determined on the basis of the unique number (Unique Identifier, UID) that is recognized at fixed zones equipped with the corresponding RWDs. Moreover, in the case of using RFID transponders-sensors, it is possible to gather data about environmental parameters prevailing during movement/transport/disposal. Thus, the greater the density of RWD antenna network is, the greater the location accuracy can be obtained. However, the cost of such an installation containing multiple RFID devices is disproportionately high compared to other known localization methods. Therefore, the determination of objects position is usually limited only to indicate a room (access to rooms, e.g., using NFC). Nevertheless, it should be emphasized that costs of passive transponders are very low in relation to short range devices (SRD) and it is easy to integrate them with different kind of products/things.

The problem of using the RFID technology in localization and navigation of moving objects, particularly in buildings and in areas with the dense urban infrastructure, is a common topic of Research and Development (R&D) projects [114,115]. Some works are carried out towards estimating position of objects marked with transponders, by analyzing parameters of electromagnetic waves received by RWD antennas deployed at various points in a room. In such solutions it is required to use advanced algorithms in processing of collected data in order to estimate the quality of received signals. For example, it is possible to analyze the Received Signal Strength Indicator (RSSI) factor which value decreases with the increase in distance between the interrogator and the transponder [116]. A phase (Angle of Arrival, AOA) [117] or a delay (Time of Arrival, TOA) [118] of the feedback signal can be a similar determinant of the distance. For example, in the LANDMARC system, the signal strength (RSSI) registered from the transponder located on a tagged object is compared to corresponding values obtained for signals originating from reference points deployed in the working area—the object location is determined on the basis of the comparison result [119]. It is important that all transponders have to be identical and, moreover, they have to operate under the same environmental conditions, what is difficult to achieve in practice.

Triangulation methods are also known in the localization technique in which the RFID technology is used [114]. They are based on analyzing the response coming from the transponder attached to a moving object and received by the multiple RWDs placed at various workspace points. In this case, due to the operating principles and specific construction of the passive RFID transponders, the obtained accuracy of the localization strongly depends on the propagation conditions of radio waves prevailing in the interrogation zone. 

**Figure 29:**
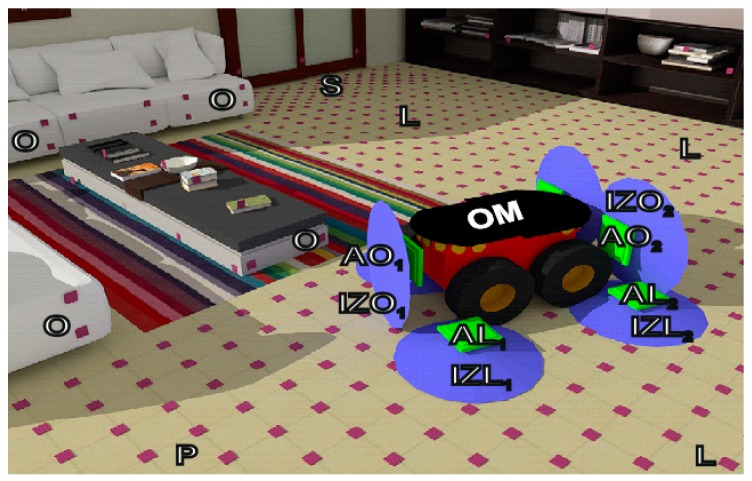
Idea of intelligent navigation system. P—navigation network; L—area with standard point density; S—area with changed density of points; O—marked obstacles; OM—mobile object; AL_n_—RWD antenna; IZL_n_—interrogation zone.

Searching for new applications of the RFID technology, the authors also focused on developing navigation systems for mobile objects. In considerations presented in [120], absolute location of the object in a given area (e.g., based on the recognized UID code of the transponder placed at a known point in a room) is not as essential as making navigation decisions. The direction of the movement is dynamically created in the intelligent center at the RWD site on the basis of the map of reference points. The key of the presented idea is to deploy transponders in the working area in such a manner that allows keeping navigations and recognizing obstacles (type of floor covering, permanent furnishings, structural elements or furniture in a room) (Figure 29) at the lowest cost of the system. 

**Figure 30:**
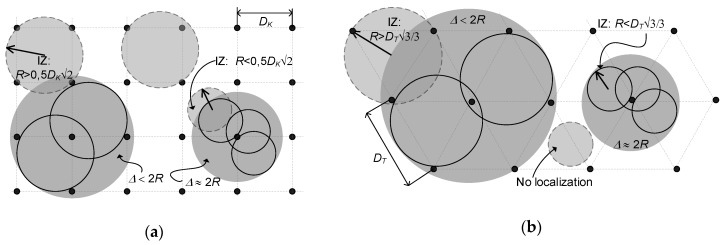
Considered distributions of RFID transponders/sensors: (**a**) Square network; (**b**) Triangle network. *D_K_*, *D_T_*—network constant for square and triangle meshes, respectively; *Δ*—localization area; *R*—radius of IZ; ⚫—RFID tag, node of network.

In the case of using RFID sensors, it is also possible to monitor the current ambient conditions (temperature, humidity, substrate roughness) and make the decision to continue or change the object movement direction, increase or decrease its speed, etc. The extended memory in the RFID chip can be also used for providing additional navigational information, e.g., on the temporary condition of a surface, changes in obstacle location or frequency of using a communication path. Since the network of transponders has the ability to saving multitude data, a natural structure is created to build fully autonomous systems where the mobile object can independently search the way to the assumed destination, build a map of available areas and permissible traffic parameters, mark already recognized paths, etc. The accuracy of the object localization and its trajectory determination depend mainly on the density of transponder network nodes and the size of the interrogation zone. The both parameters were analyzed in [120]. The IZ parameter can be changed dynamically, e.g., by adjusting the power supplied to the RWD antenna or in more advanced solutions, by implementing antenna system with controlling phase of signals [121,122]. In turn, the network of identification points can have different density and shape as well as it can be regular or chaotic. In order to carry out the analysis, two exemplary ways of placing reference points are proposed: square network and triangular network (Figure 30).

The highest accuracy of the localization (Figure 30) is obtained when 4 (for square network) or 3 (for triangular network) UID numbers are simultaneously recognized and then respectively:

(18) $$R=0.5D_{K}\sqrt{2}\mathrm{for}\;\mathrm{square}\;\mathrm{network},$$

(19) $$R=0.5D_{T}\frac{\sqrt{3}}{3}\mathrm{for}\;\mathrm{triangular}\;\mathrm{network}.$$

The reading data from several transponders/sensors has to take time *t_S_* (21), and therefore it limits the speed of supervised object movement. In turn, reduction of the IZ area size can lead to the situation that no network node will be in range, so the direct indication of position is not possible—it is when:

(20) $$R<0.5D_{K}\sqrt{2}\begin{array}{ccc} & \mathrm{or} & \end{array}R<0.5D_{T}\frac{\sqrt{3}}{3}.$$

In this case the location can be estimated on the basis of software algorithms, until the next transponder/sensor is encountered. 

The time that is necessary to recognize UID, read and write the transponder memory depends on the type of RFID system commands that have to be carried out in the telecommunication protocol:

(21) $$t_{s}=t_{AC}+\sum_{1}^{k}\{t_{Pk},t_{READ}{}_{k},t_{WRITE}{}_{k},\ldots \}$$

where *k* indicates subsequent transponder, *t_AC_*—duration of anti-collision procedure, *t_Pk_*—time necessary to perform data acquisition in case of using non-autonomous RFID sensors, *t_READk_*—time spend on reading *k*-th transponder, *t_WRITEk_*—time spent on writing *k*-th transponder. By modifying and coding some data, the *t_S_* parameter can be significantly shortened [120]. Determination of the traffic path in the intelligent system can be based only on memorizing and searching for the sequence of numbers assigned to the nodes, instead of recognizing the multi-byte UID. There is not always a need to read the contents of the entire chip memory. By entering a byte of flags, it can be easily indicated which data has to be read and which can be omitted. The shorter the time is, the higher the speed of moving object can be obtained. 

**Figure 31:**
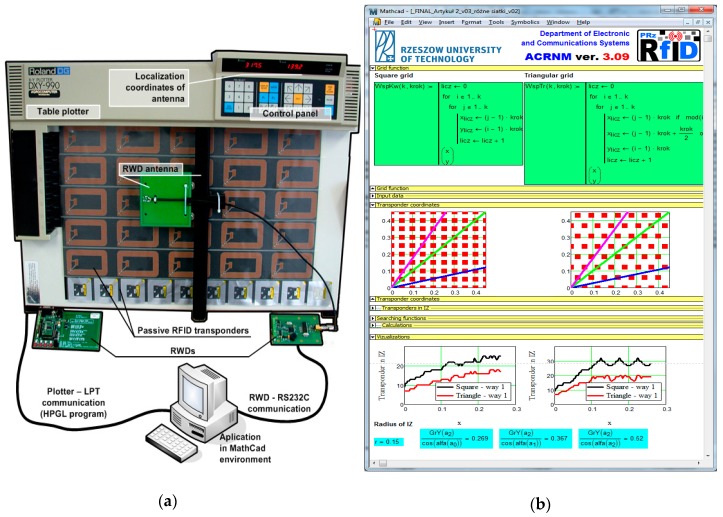
Laboratory stand for testing autonomous navigation system in scope of configuration network of reference points: (**a**) Hardware; (**b**) Software tool in Mathcad environment.

In order to confirm the correctness of established assumptions, a special test stand was prepared (Figure 31). It is built with a common XY plotter in which an RWD antenna is attached to a movable arm and nodes are made of transponders working in the HF band. Both the plotter (arm position) and the read/write devices (output power regulation, data acquisition) are controlled from the computer system.

A system of autonomous localization and collision-free movement of mobile object in the space with obstacles that is based on the RFID technology is the subject of patent PL 220427 B1 issued by the Polish Patent Office [123]. The main reservations concern the method of deploying network reference points according to the required accuracy of the localization, marking obstacles (with additional transponders or by recording appropriate warnings and obstacle characteristics in transponders of the basic network), sharing supplementary navigational information (i.e. surface parameters, terrain configuration). In an environment equipped with such a system, the mobile object can move fully autonomously recognizing surroundings and building its own map in a dynamic way. Therefore, there is no need to use an additional supervising computer center and sets of motion paths as well as to store a full map of transponder distribution. Detection of a transponder associated with the obstacle allows to get complete information about shape of encountered item and how to overcome it. Once recognized path can be marked by appropriate writings in the chip memory and then it can be crossed with greater dynamics (by ignoring the extended information and thus reducing the *t_S_* time). The saved information can also be used in the collision avoidance process—by maintaining extreme caution when crossing the communication paths used by other mobiles. The invention is dedicated for using primarily in industrial halls, warehouse facilities, large-surface stores but can also be utilized in residential or office buildings when controlling robots or devices that support the activity of disabled people, etc.

**Figure 32:**
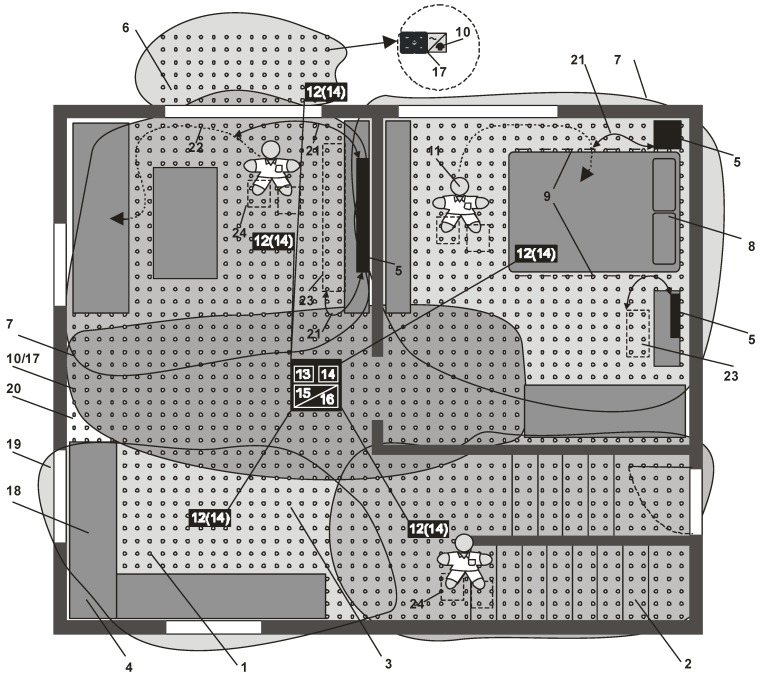
Idea of the active floor: 1—operating area; 2—stairs; 3—room; 4—building equipment elements; 5—controlled electrical and electronic devices; 6—outside area; 7—IZ; 8—possible object position outside operating area; 9—boundary line of the operating area that triggers action in system; 10—node of measuring network; 11—monitored object with specific weight; 12—antenna; 13—multi plexer; 14—RWD (optional occurrence in parentheses); 15—PC; 16—software; 17—passive RFID transponder-sensor; 18—area excluded from object movement, does not belong to operating area; 19—IZ outside operating area (no node points); 20—area with node points but outside operating area (no coverage by IZ); 21—personalized reaction of control system; 22—path of object movement; 23—activation area; 24—activated area.

The continuation of works in design and implementation of the RFID sensors led to the preparation of the next invention. Its subject is the active floor (Figure 32) and the personalized control system using an active floor, particularly applicable to control of electrical and electronic devices in intelligent buildings as well as object movement monitoring. The invention is based on RFID system operating in the UHF band with autonomous battery-free RFID sensors hidden in the floor cladding. The overall concept consists in recognizing objects and their position (e.g., a person, a mobile robot) by measuring the pressure force applied to the substrate. Actions adapted to the personal requirements classified by the weight are taken on the basis of data read by the RWDs and processed in the control system. Some modifications of the concept allow further extension of the invention’s potential.

For example, the location and personalization process can be based on RFID detectors (instead of a weight measurement, only a presence detector can be used) and object recognition can be implemented based on biometric features (speed of movement, step length, etc.) using software algorithms. The activations of transponders only after load change detection (which significantly reduces the load on the communication link), integration with various substrate types in an aesthetic and invisible way, tracking of relocated items (resulting from monitoring the weight changing at the sensor nodes or recognizing transponder integrated with object), connection with typical home automation and alarm systems, use of transponders integrated with the floor covering throughout its life cycle (from production to utilization, which can significantly reduce the system implementation costs) as well as elimination disturbances resulting from natural use, substrate aging, ambient conditions (temperature, humidity, etc.) are other modifications of the idea. Additional application areas may concern security systems, integration with services added to smartphones, Internet of things, social care homes, rehabilitation centers, hospitals where there is a need of monitoring patient activity, etc.

## 13. Summary

An attempt to systematize the process of synthesizing autonomous sensors with RFID interface has been presented in this paper. The authors have tried to point out problematic cases in many practical and theoretical studies that arise when creating new RFID device constructs. They have revealed the vagueness of producers’ datasheets, the lack of complete characteristics and some important parameter values of offered products as well as incompleteness of the knowledge regarding the practices of designing RFID systems. To deal with this problem, they have presented several useful procedures for measuring important parameters and have elaborated dedicated methods and lab benches stands to support the characterization of the components used in RFID technology. Further, to cope with the encountered inconveniences, they have worked out an experimental board devoted to developing battery-less autonomous semi-passive RFID transponders in which extra functions of energy harvesting from the electromagnetic field of different radiocommunications systems, the power conditioner and storage block of ultra-low energy are implemented. The proposed system and procedures are available and can be used by other designers who want to create new implementations of RFID sensors. In this way, the authors want to change widespread practice of employing “trial and error method” that is commonly used when new RFID applications are developed.

The proposed concepts of the autonomous sensor with the RFID interface fits in the forecasts for developing the systems of automatic object identification. According to the assumed classification, well-known bar codes are included in the first basic stage [23]. Due to their significant limitations (single identification, the need of presenting the code in the field of view of an optical reader, limited amount of coded information), they have ceased to meet the needs of the product and service market. Therefore, solutions based on the RFID technology which are classified to the second stage of the mentioned developmental model are appeared more and more often. An unquestionable advantage in this case is the ability to conduct the anti-collision identification process without the need to provide optical access to the object. Moreover, much larger amount of data placed in the semiconductor memory can be read as well as modified according to user application preferences. Furthermore, beside detailed information about the marked object also measurement data obtained from the environment by the built-in sensors of various physical quantities can be stored in the chip memory. This functionality gains advantages of RFID technology and allows to create construction that move autonomous identification system to the next—third stage of the development classification.

The need and the possibility of designing RFID sensors was noted at the stage of preparing the patent application [103,123] and the idea was further developed in subsequent publications and projects, including primarily grants financed by the Polish government and European Union. The obtained results were implemented in the work carried out for entrepreneurs, mainly in the industrial research performed for the Intelligent Development Operational Program, aimed at developing a semi-passive RFID transponder-sensor for using in management system that includes production, distribution, installation, operation, service/maintenance and utilization of photovoltaic panels. Currently, research on factors affecting the synthesis of RFID transponders is continued and achieved comprehensive development of authors’ scientific experience takes special meaning in the context of inability to develop universal design of the autonomous RFID sensor that could be implemented in any application of automatic object identification system. Since the trial and error method is most often used in the common practice of designing new RFID devices and majority of parameters are only estimated based on the tests and knowledge of designer teams, the presented paper should contribute to some progress in the RFID technology.

## Figures and Tables

**Table 1:** Selected parameters of commercial chips dedicated to semi-passive RFID transponders of the UHF band.

No.	Parameter	SL900A ^4^	ROCKY100 ^5^	EM4325 ^6^	WM72016-6 ^7^
1.	Operating frequency (MHz)	860–960	860–960	860–960	860–960
2.	EPC compliance	C1/3 G2	C1 G2	C1/C3 G2	C1 G2
3.	ISO/IEC compliance	18000-63	18000-6c	18000-63 18000-64	18000-6c
4.	Battery support (V)	1.5 or 3	1.4–3.0	1.25–3.6 ^1^	2.1–3.0
6.	Memory capacity (kb)	9	~1	4	16
7.	EPC/TID (b)	144/80	128/48	352/48	96/64
8.	User memory (b)	8416	1008	3072	~16000
9.	Temperature of operation (°C)	−40–125	−40–85	−40–85	−40–85
10.	Temperature sensor (°C)	Inter. −20–60 ^2^	---	Inter. −40–60	---
11.	Digital interface	SPI ^3^	SPI	SPI	Dual SPI

^1^ Min. 1.8 V for writing to EEPROM; ^2^ For external sensor: −140–125 °C; ^3^ Additional analog inputs to analog-to-digital converter (the possibility of connecting two additional sensors, including a temperature sensor with a range of −40–125 °C); ^4^ https://ams.com/SL900A; ^5^ http://www.farsens.com/en/products/rocky100/; ^6^ https://www.emmicroelectronic.com/product/epc-and-uhf-ics/em4325; ^7^ https://www.cypress.com/file/120726/ download.

**Table 2:** Impedance of ink-jet printed flexible antenna dedicated to the UHF band.

	Model ^1^	Model ^2^	S.#1	S.#2	S.#3	S.#4	S.#5	S.#6	S.#7
**Re(*Z*) (Ω)**	37	51	129	130	114	113	115	110	107
**Im(*Z*) (Ω)**	336	479	433	455	440	447	447	431	449

^1^ Model of the antenna placed in the free space; ^2^ Model of the antenna attached to the PET bottle.

**Table 3:** Impedance of ink-jet printed flexible antenna dedicated to the HF band.

	Model	S.#1	S.#2	S.#3	S.#4	S.#5	S.#6	S.#7
***R_TS_* (Ω)**	39.3	46.5	39.5	35.8	35.3	35.5	41.0	51.1
***L_TS_* (µH)**	5.04	5.50	5.52	5.48	5.46	5.49	5.48	5.51

**Table 4:** Impedance of PCB antenna dedicated to the HF band.

	Model	S.#1
***R_TS_* (Ω)**	8.59	8.68
***L_TS_* (µH)**	5.08	5.10

**Table 5:** Examples of research results: chip sensitivity vs. frequency and *U_bat_*.

*f*_0_ (MHz)	*P_Tmin_* (dBm) at *U_bat_* = 0 V		*P_Tmin_* (dBm) at *U_bat_* = 1.5 V	
	Method (a)	Method (b)	Method (a)	Method (b)
866	−13.1	−13.1	−14.8	−14.8
900	−13.7	−13.6	−15.2	−15.1
915	−13.4	−13.4	−14.9	−15.0
860–960	−13.2	−13.1	−14.9	−14.8

**Table 6:** Examples of research results: read range vs. frequency and *U_bat_*.

*f*_0_ (MHz)	*r_Max_* (m) at *U_bat_* = 0 V	*r_Max_* (m) at *U_bat_* = 1.5 V
866	4.08	4.96
900	4.21	5,00
915	4.00	4.75

**Table 7:** Comparison of measurement and calculation results of *r_Max_* for a RFID sensor for different loads *P_TH_* of internal energy harvester.

*P_TH_* (mW)	Model *H_min_* (A/m)	Measurement *H_min_* (A/m)	*r_Max_* (m)
0	0.028	0.027	0.6
1	0.120	0.123	0.35
4	0.461	0.462	0.19

**Table 8:** Comparison of measurement and calculation results for IZ boundaries.

	Step #1	Step #2	Step #3	Step #4	Step #5	Step #6	Step #7
***r_ID_* (m)**	0.15	0.20	0.25	0.30	0.35	0.40	0.45
**Model: *b* (m)**	0.30	0.31	0.33	0.30	0.26	0.20	0.14
**Measurement: *b* (m)**	0.28	0.30	0.32	0.30	0.25	0.19	0.14
